# Linkage Progression Mapping: Precision Structure Analysis of Individual Oligomers in Birch Milled Wood Lignin

**DOI:** 10.1021/acs.biomac.5c02761

**Published:** 2026-03-30

**Authors:** Filippa Ludvig, Åsa Emmer, Martin Lawoko

**Affiliations:** † Wallenberg Wood Science Centre, Royal Institute of Technology, KTH, 100 44 Stockholm, Sweden; ‡ Division of Wood Chemistry and Pulp Technology, Department of Fibre and Polymer Technology, School of Engineering Sciences in Chemistry, Biotechnology and Health, KTH Royal Institute of Technology, 100 44 Stockholm, Sweden; § Analytical Chemistry, Applied Physical Chemistry, Department of Chemistry, School of Engineering Sciences in Chemistry, Biotechnology and Health, KTH Royal Institute of Technology, 100 44 Stockholm, Sweden

## Abstract

Molecular heterogeneity in lignin remains poorly understood due to the lack of analytics robust enough to determine the precise structure of individual molecules in isolated lignin samples. Recently, we showed that MALDI-TOF MS analysis, combined with a range of NMR techniques, facilitated comprehensive structural studies of oligomer populations present in milled wood lignin from spruce, without prior fractionation. Here, the developed methodology is applied to study populations in milled wood lignin from birch. We report a dominance of linear aryl ether homo-oligomers subdivided into three categories: oligomers exclusively of sinapyl units, oligomers exclusively of guaiacyl units, and oligomers with both guaiacyl and sinapyl units. Linkage progressions in oligomeric structures with the other common interunits are also elucidated. Unlike previous reports, the study enabled the determination of molecule-specific syringyl/guaiacyl ratios. All of the elucidated oligomers contain enone or enal groups at the aliphatic end and phenolic hydroxyls on the other, highlighting homolytic cleavage reactions that occur during lignin procurement. Overall, the study provides a comprehensive framework for an atomistic understanding, offering significant potential for both fundamental and applied research.

## Graphical abstract

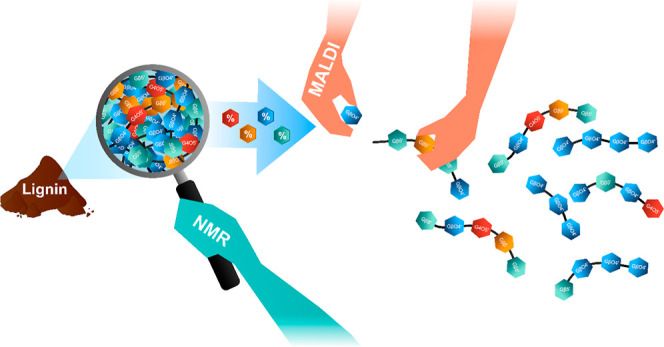

## Introduction

Woody biomass is composed of three major components: cellulose, hemicellulose, and lignin.^1^ While there is extensive structural information available on both cellulose and hemicelluloses, lignin remains analytically elusive and is typically represented in the literature by a global structural motif.^2^ In hardwoods, lignin is mainly derived from two precursors: sinapyl- and coniferyl-alcohol. The radical coupling reaction during polymerization yields a variety of different interunit linkages, with the most common being aryl ethers (βO4′), phenylcoumaran (β5′), and pinoresinol (ββ′).^1,3,4^

Softwoods consist almost entirely of guaiacyl (G) units, while sinapyl (S) units dominate hardwoods despite also containing significant amounts of G-units (Figure 1).^3^ The term S/G ratio is often used to express the relative monomeric composition of hardwood lignin. S/G ratios of around 2–3 have been reported for hardwoods in the literature;^5–7^ it should be noted, however, that the reported S/G ratios are averages, as opposed to being molecule-specific. Thus, the determination of S/G ratios in a molecularly resolved manner would be interesting for both fundamental and applied research.

**Figure 1:**
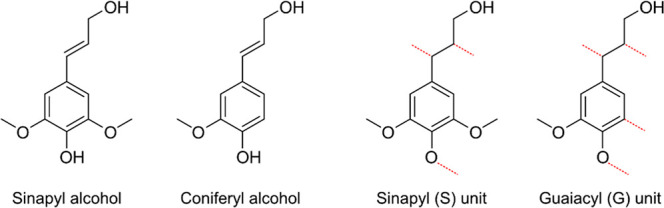
Structures of sinapyl- and coniferyl-alcohol as well as the possible bonding sites when incorporated as S- and G-units in lignin.

While state-of-the-art techniques, such as nuclear magnetic resonance (NMR) spectroscopy, have been successfully used to study interunit linkage types, the determination of linkage progression in individual oligomers is scanty. Previous studies have utilized various MS and MS^*n*^ systems to identify, evaluate, and sequence lignin model compounds and isolates.^8–15^ While structural identification in previous literature has been successful and several key mass increments in MS and fragmentation pathways in MS^*n*^ have been identified for the most common bonds,^8–10,13,14,16,17^ complex MS^2^ fragmentation patterns on nonmodified lignin oligomers complicate interpretation and do not always provide facile analysis. Nevertheless, mass spectrometry-based sequencing has been used on lignin oligomers in Poplar to afford complete sequencing of 36 trimers to hexamers, where collision-induced dissociations (CID) of phenoxide anions played a key role in deciphering the fragmentation patterns and cluster formations.^8,9^ Computational methods to interpret and showcase information, such as Van Krevelen plots, Kendrick mass defect plots, and principal component analysis, on data sets gathered from high-resolution MS techniques have proven useful for molecular characterization of various lignins as well as depolymerization products.^14,18–21^

Using matrix-assisted laser desorption/ionization time-of-flight mass spectrometry (MALDI-TOF MS), a soft-ionization MS technique, we recently showed that acetylating lignin was sufficient to study intact interunit linkage progressions in milled wood lignin (MWL) from softwood.^22^ The discernment principle is based on the fact that the most commonly occurring bonds in lignins have a unique number of hydroxyl groups in the aliphatic side chain, which upon acetylation will yield a unique mass increment for each formed interunit type. The unique mass increment from bond formation can therefore be used to fingerprint the specific interunits added to the growing lignin molecule by endwise addition, hence providing the interunit linkage progression. This concept is further developed here in the Results section.

For larger and more complex lignin oligomers, there is still a need for the development of robust and facile analytical methodologies for the analysis of individual molecules. Furthermore, simplifying the sequencing of lignin by bypassing tedious interpretation of intricate MS^*n*^ fragment spectra would contribute to greatly improved characterization of this complex class of biomolecules. Hence, we believe that the proposed framework in this study provides advantages such as simplicity, minimal sample preparation, and facile data interpretation when compared to the earlier mentioned established MS-based methods.

In this work, we study Birch MWL using our recently developed methodological framework.^22^ Specifically, the aim is to elucidate lignin linkage progressions, determine precise structures of individual molecules, and provide insights into molecularly resolved S/G ratios in hardwood birch. Adding value to the lignin analytics field, this will provide new insights into lignin heterogeneity, specifically tied to S/G ratios of individual molecules, how both the monomers and the interunit linkages are distributed, and whether there are any correlations between these. This is primarily investigated by using MALDI-TOF MS on acetylated birch MWL. To validate the constructed linkage progression map (LPM), laser-induced dissociation (LID) MALDI-TOF MS^2^ was applied to determine the structures correlated with selected adduct peaks in the MALDI-TOF spectra. On the basis of the fragmentation patterns, it is suggested that all of the investigated acetylated oligomers possess the same modified aliphatic end unit, which contains an enone or enal moiety. In some cases, MS^2^ investigations imply an overlap of different oligomers at the same *m*/*z,* highlighting the heterogeneity of lignins and underscoring the importance of developing methodologies that allow for an atomistic understanding of individual molecules.

## Methodology

### Chemicals

*N*,*N*-Dimethylformamide (DMF) (anhydrous) 99.8%, pyridine (anhydrous) 99.8%, CDCl_3_-*d* ≥ 99.8 at. % D, endo-*N*-hydroxy-5-norbornene-2,3-didicarboximide (eHNDI) 97%, chromium­(III) acetylacetonate (Cr­(AcAC)_3_) 99.99%, 2-chloro-4,4,5,5-tetramethyl-1,3,2-dioxaphospholane (CI-TMDP) 95%, acetic anhydride ≥99%, 2,5-dihydroxybenzoic acid (DHB) for MALDI-MS ≥ 99%, trifluoroacetic acid 99%, dimethyl sulfoxide-*d*_6_ 99.9% D, and toluene ≥99.5% were obtained from Sigma-Aldrich. Acetonitrile ≥ 99.9% was obtained from Honeywell Riedel-de Haën. MeOH 99.8% was obtained from VWR Chemicals. Milli-Q was obtained from a Millipore Ultrapure System equipped with a MilliPak 0.22 μm filter. α-Cyano-4-hydroxy-cinnamic acid (HCCA) for MALDI-TOF MS was obtained from BRUKER Daltonik GmbH. Milled wood lignin from Birch obtained by the Bjorkman procedure^30^ was a kind gift from the laboratory of Dr. Nilvebrant at Borregaard.

### Methods

Acetylation of Birch MWL follows a previously established protocol.^31^ Glassware for acetylation was dried in an oven (105 °C, overnight). Pyridine and acetic anhydride were mixed (1:1 v/v) and added to the round-bottom flask. Lignin was added while stirring. The round-bottom flask was covered in aluminum foil and left to react (RT, overnight). The reaction was quenched with MeOH (30 min) in an ice bath. Toluene was added portion-wise and rotary-evaporated to yield the acetylated lignins. The acetylated sample was further dried under a stream of N_2_ to yield the final derivatized product. ^31^P NMR and MALDI-TOF MS^*n*^ analysis were carried out on the acetylated sample.

For MALDI-TOF MS analysis, the nonmodified and the acetylated lignin were dissolved (2.4 mg mL^–1^) in DMF. 2,5-DHB was prepared at 20 mg mL^–1^ in TA50 (50:50 ACN:0.1% TFA). The sample solution (0.5 μL) was applied onto an Anchorchip MTP 384 Target Plate and dried under a stream of N_2_. The 2,5-DHB matrix solution (0.5 μL) was applied on top of the dried sample and consequently air-dried. Three replicates were made for each sample. Spectra were acquired on a BRUKER ultrafleXtreme (Bruker Daltonics, Bremen, Germany) using an Nd/YAG laser (355 nm) in reflectron mode. The mass spectrometer is calibrated using a Bruker Peptide Calibration Standard for external calibration in positive mode. For each spectrum, 4000 (4 different locations on the sample spot × 1000 shots on each location) laser shots were used for the generation of one spectrum in the 0–5000 *m*/*z* region in positive mode. The data was acquired using flexControl 3.4, and processed using flexAnalysis 3.4.76. Mass lists were generated using the “*centroid*” function to identify peaks.

For MALDI-LIFT-TOF-TOF MS^2^, the same acetylated lignin sample solution (2.4 mg mL^–1^ in DMF) used for MALDI-TOF MS was used. Saturated HCCA in TA50 was prepared, with a visible pellet at the bottom. The HCCA solution was briefly centrifuged to spin down nonsolubilized particles. The sample solution (0.5 μL) was applied onto an Anchorchip MTP 384 Target Plate and dried under a stream of N_2_. The HCCA solution (0.5 μL) was deposited on top of the dried sample spot and allowed to air-dry. Three replicates were made. Parent spectra were collected with a laser beam attenuation of 43, a laser beam focus of 70, and a reflector detector voltage of 2.107 kV. Fragment spectra were collected with a laser beam attenuation of 20.2, a laser beam focus of 70, and a reflector detector voltage of 2.212 kV. The LIFT voltage 1 was 19 kV, and the LIFT voltage 2 was 3.7 kV. The reflector voltage 1 was 29.5 kV, and the reflector voltage 2 was 14 kV. Parent and fragment spectra were saved as one, merging the spectra together as a total of 20.000 laser shots. Parent spectra were collected using 8.000 laser shots, and fragment spectra were collected using 12.000 laser shots. Data was acquired using flexControl 3.4 and processed using flexAnalysis 3.4.76. Mass lists were generated using the “*centroid*” function to identify peaks.

^31^P NMR on nonmodified and acetylated lignin was performed on a Bruker Avance III HD 400 MHz (Bruker Corporation, Massachusetts, USA) instrument, with a BBFO probe with a Z-gradient coil (5 mm PABBO BB/19F-1H/D Z-GRD Z116098/0174). A previous protocol has been followed.^32,33^ The internal standard (eHNDI) (30 mg) was pipetted to pyridine (500 μL). Cr­(AcAc)_3_ (approximately 3 mg) was added to the internal standard solution. Lignin (29.9 mg) was weighed. Pyridine (100 μL) and DMF (100 μL) were added to the lignin, and the solution was vortexed until complete dissolution. The eHNDI solution was added (50 μL) to the lignin solution and vortexed. 2-Chloro-4,4,5,5-tetramethyl-1,3,2-dioxaphospholane (100 μL) and CDCl_3_ (450 μL) were added dropwise to the sample mixture. The reaction was allowed to proceed for 30 min before the solution was transferred to an NMR tube using a Pasteur pipet. The NMR method “N P31ig (P31 for lignin)” was used. Number of scans: 256. 5s relaxation delay. MestReNova software (Mestrelab Research) was used for the analysis of the spectrum.

2D-HSQC NMR and HMBC NMR were performed using a Bruker 400 DMX (Bruker) instrument, equipped with a 5 mm probe (PA BBO 400S1 BBF-H-D-05 Z) at 298.0 K. Spectra were analyzed with MestReNova software. Data processing included Fourier transformation, phase, and baseline correction in both dimensions using a third-order Bernstein polynomial fit. The C2–H region was used as an internal standard. 80 mg of the sample was prepared in 800 μL of DMSO-*d*_6_.

HMBC spectra were obtained with the pulse program “hmbcgpl2ndqf”. 200 scans over 512 × 256 increments, 1.5000 s relaxation delay, and 0.1065 s acquisition time.

^1^H ^13^C HSQC spectra were obtained with the pulse program “hsqcetgpsi2”. 128 scans over 512 × 256 increments, a 2.0000 relaxation delay, and a 0.1065 acquisition time.

## Results and Discussion

We will first show the importance of NMR in the determination of lignin interunits and then demonstrate how it supports the linkage progression mapping performed by MALDI-TOF MS analysis to yield oligomers with well-defined structures. We then validate the detailed structural features of ten selected oligomer peaks by utilization of MALDI-TOF MS^2^.

### NMR Analysis of MWL

The 2D-HSQC NMR analysis confirms the presence of the well-established interunit linkage types in Birch MWL (Figure 2B) with the most common being βO4′, ββ′, and β5′ (structures given in Figure 2A). A high content of βO4′ at 52% is obtained, which is consistent with already established literature,^3^ while the contents of ββ′ and β5′ are semiquantified to 7% and 2%, respectively (Figure 2C). Moreover, weak carbohydrate signals are observed (Figure 2C). Aldehydes (Figure 2B*) are also detected. The approximate S/G ratio, as determined from the 2D-HSQC, is 2.3 (Figure 2) and is consistent with the established literature on Birch MWL.^5,6^

**Figure 2:**
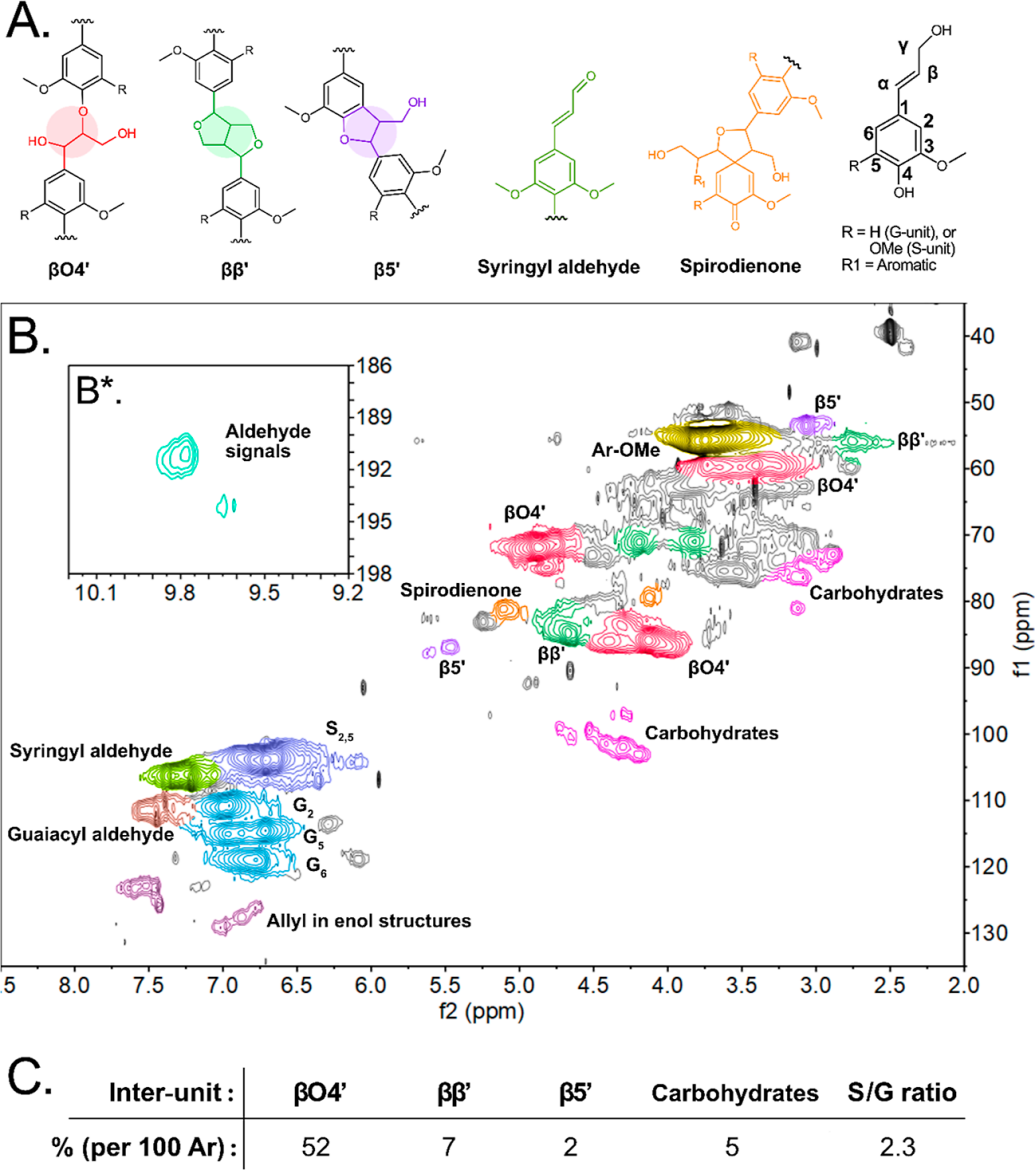
^13^C ^1^H 2D-HSQC NMR of Birch MWL. The inset shows aldehyde-correlated peaks {^13^C: 186–198 ppm, ^1^H: 9.2–10.2 ppm}. (A) Structures of interunit linkages identified in spectra, left to right: βO4′, ββ′, β5′, syringyl aldehyde, spirodienone, and a general schematic for carbon nomenclature used. (B) 2D-HSQC spectra, with the insert (B*) of signals found in the aldehyde region. (C) Semiquantification of groups in the sample, given as a relative percentage per 100 aromatic units. Full HSQC spectra can be found in Figure S1.

In our previous work on MWL from spruce, enals and enone end groups were found to be crucial molecular anchors for linkage progression studies by MALDI-TOF.^22^ They were proposed to originate, in part, from the homolytic cleavage reactions of aryl ethers resulting from the mechanical energy used in MWL isolation. Thus, for the sake of MALDI-TOF analysis, we investigate the presence of these crucial structures by heteronuclear multiple bond correlation (HMBC) 2D-NMR (Figure 3).

**Figure 3:**
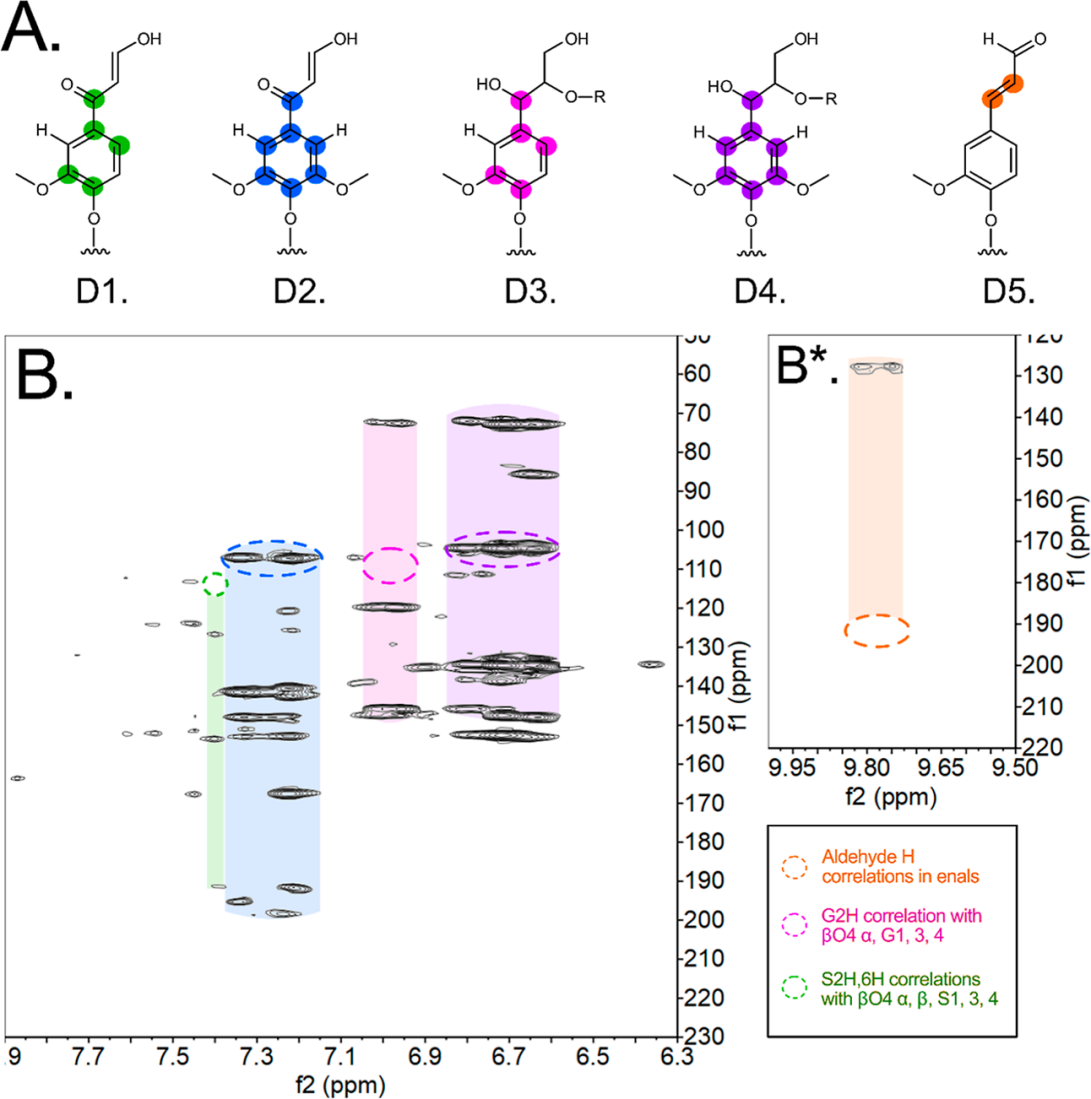
HMBC NMR of nonmodified Birch MWL. (A) The relevant protons for the through-bond atom connectivity studies are shown, and the respective carbons are marked. (B) Aromatic region. (B*) Aldehyde region. Bottom right denotes the marking used to explain identification and correlation.

The complete HMBC spectrum is presented in Figure S2, and relevant sections are highlighted in Figure 3 for further discussion. The oxygenated aliphatic regions (^1^H: 3–5.5 ppm, Figure S2) show weak signals, with only methoxy- and βO4′ proton correlations visible. Correlations of ββ′ and β5′ protons are likely suppressed to the noise level due to the relatively lower concentrations as seen in the HSQC (Figure S1).

The aromatic region (^1^H: 6–8 ppm) in Figure 3B shows several aromatic proton correlations. For instance, correlations of the H_2_ aromatic proton in G-units with the α-carbon in the βO4′ structure are seen (color-coded pink). Similarly, correlations of the H_2,6_ aromatic proton in S-units with α- and β-carbons in βO4′ can be seen (purple color-coded). Correlations of G- and S-protons (specifically G-H_2_ and S–H_2,6_) in enone structures are also suggested (color-coded green and blue, respectively). Furthermore, the protons of different aldehydes (^1^H: 9.7–9.9 ppm) correlate with allylic carbons, suggesting enal structures (color-coded orange). Both enones and enals have been proposed in our recent studies on Spruce MWL.^22^ These structures will be of interest in elucidating linkage progressions by MALDI-TOF MS.

### Linkage Progression Mapping by MALDI-TOF MS Analysis

In our recent studies of Spruce MWL, cluster analysis of MALDI-TOF spectra was used to derive the detailed structure of oligomeric lignin populations in the sample. This was possible after prescreening of interunits by HSQC NMR. Clusters of signals with distinct mass differences between them served as starting points to fingerprint the formation of specific linkages and led to the concept called the linkage progression mapping (LPM). LPM is based on the fact that lignification occurs mainly through endwise radical addition of monomers and is a continuous process. This means that several populations, with a difference in mass approximately that of a monolignol or a monolignol +18 Da, will be present in a nonmodified lignin sample. This leads to specific mass increments when certain bonds are formed. For instance, the formation of ββ′, β1′, β5′, and 4-*O*-5′ bonds, often referred to as condensed bonds, will yield the same mass increment of *m*/*z* 178 for the addition of a G-unit by endwise polymerization, or *m*/*z* 208 for the addition of an S-unit (Figure 4: B1,C1,D1,E1). The formation of an α-hydroxylated βO4′ on the other hand yields a mass increment of 196 *m*/*z* (Figure 4) due to the additional incorporation of water to the reactive quinone methide intermediate formed subsequent to the coupling of the beta and phenoxy-radicals. Thus, for the nonderivatized lignin, only the formation of an α-hydroxylated βO4′gives a unique fingerprint. The MALDI-TOF spectrum of nonmodified MWL shows only vague clustering, indicative of end-wise polymerization. Though difficult to interpret, observations are made for the nonmodified Birch MWL—such as the expected 226 increment for an SβO4′ bond formation, which is reflected in the transformations of *m*/*z* 595 to 821 *m*/*z*, 643 *m*/*z* to 869 *m*/*z*, and 925 to 1151 *m*/*z* (Figures S6 and S9). Additionally, an observed increment of 208, reflected in the transformation from 613 *m*/*z* to 821 *m*/*z*, is representative of a condensed bond formed by an S-unit, and an increment of 196 *m*/*z*, representative of a GβO4′, is observed when 909 *m*/*z* is transformed to 1105 *m*/*z* (Figures S6 and S9).

**Figure 4:**
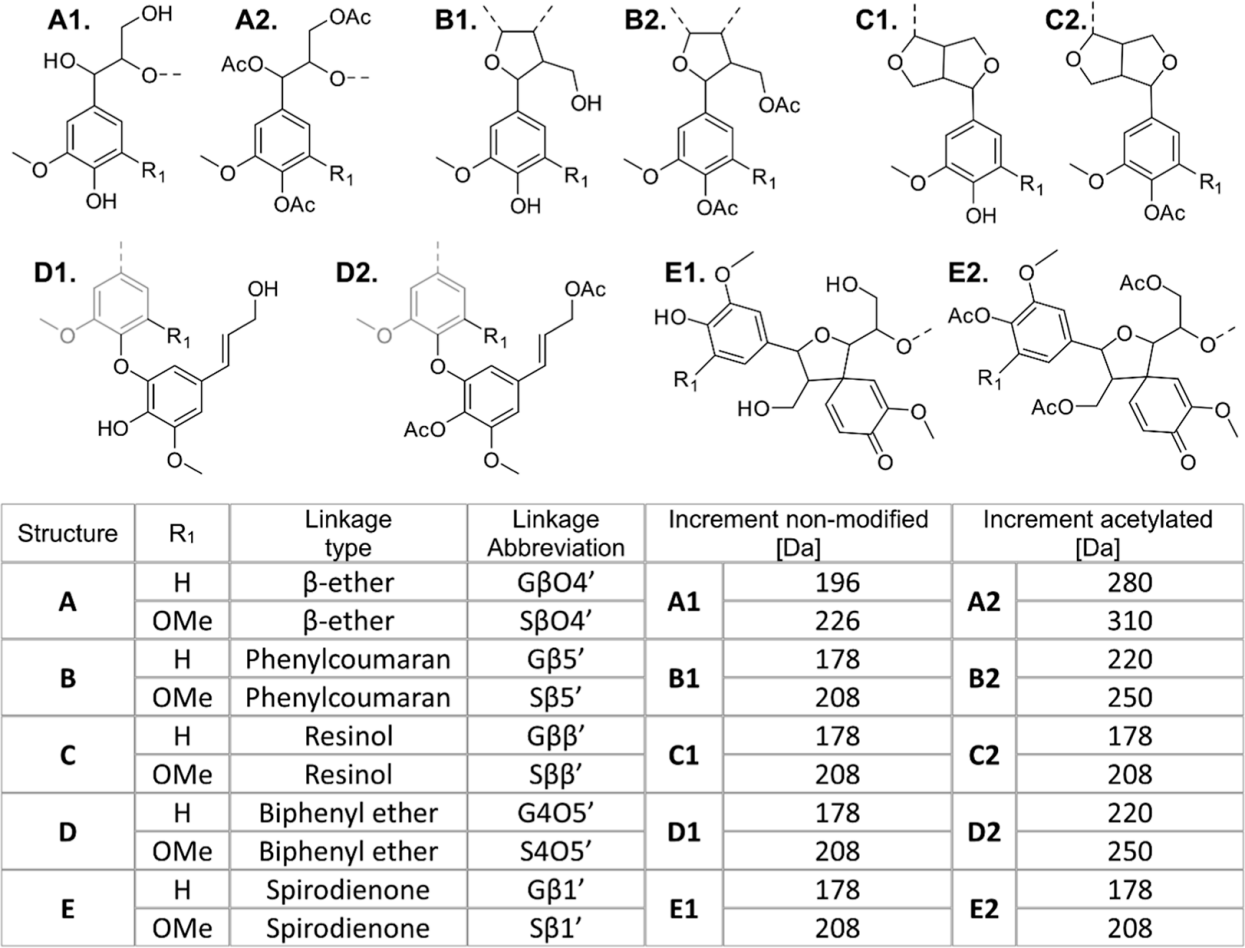
Structures for nonmodified and acetylated S- and G-units in lignin. Each structure is provided with the respective observed mass increment in MALDI-TOF MS analysis.

As mentioned earlier, the formation of the condensed bonds gives a similar increment, highlighting the need for additional strategies to distinguish between them. In this context, we show that acetylation of the MWL could be used as a means to distinguish the formation of ββ′ and β1′ from 4-*O*-5′ and β5′ bonds (Figure 4B2,C2,D2,E2). As observed, the formation of ββ′ and β1′ yields an increment of 178 for addition of a G-unit or 208 for the addition of an S unit, while the formation of 4-*O*-5′ and β5′ bonds yields an increment of 220 for addition of a G-unit, and an increment of 250 is representative of a β5′ bond from S-units. Effects of acetylation on other interunits are also indicated (Figure 4), and the resulting spectral differences from derivatization of Birch MWL can be observed in Figure 5A,B. The distinguishing principle is based on the fact that the structures have a different number of hydroxyls that can be acetylated (Figure 4). Thus, acetylation provides a high level of distinction between bonds when combined with MALDI-TOF analysis. As proof of concept, the theoretically expected mass increments resulting from fully acetylated samples are indeed found in the MALDI-TOF MS spectrum of the acetylated sample. For instance, several increments of 310, resulting from SβO4-linkage formation (Figure 4), are experimentally observed (Figure 5B*). Increments of 280 (GβO4′), 220 (Gβ5′ or G4-*O*-5′), 250 (Sβ5′), 208 (Sββ′ or Sβ1), and 178 (Gββ′ or G β1) are also found (Figure 5B*). The majority of the observed mass increments are attributed to aryl ether bond formation, which is also consistent with the HSQC results (Figure 2) and established literature.^3^

**Figure 5:**
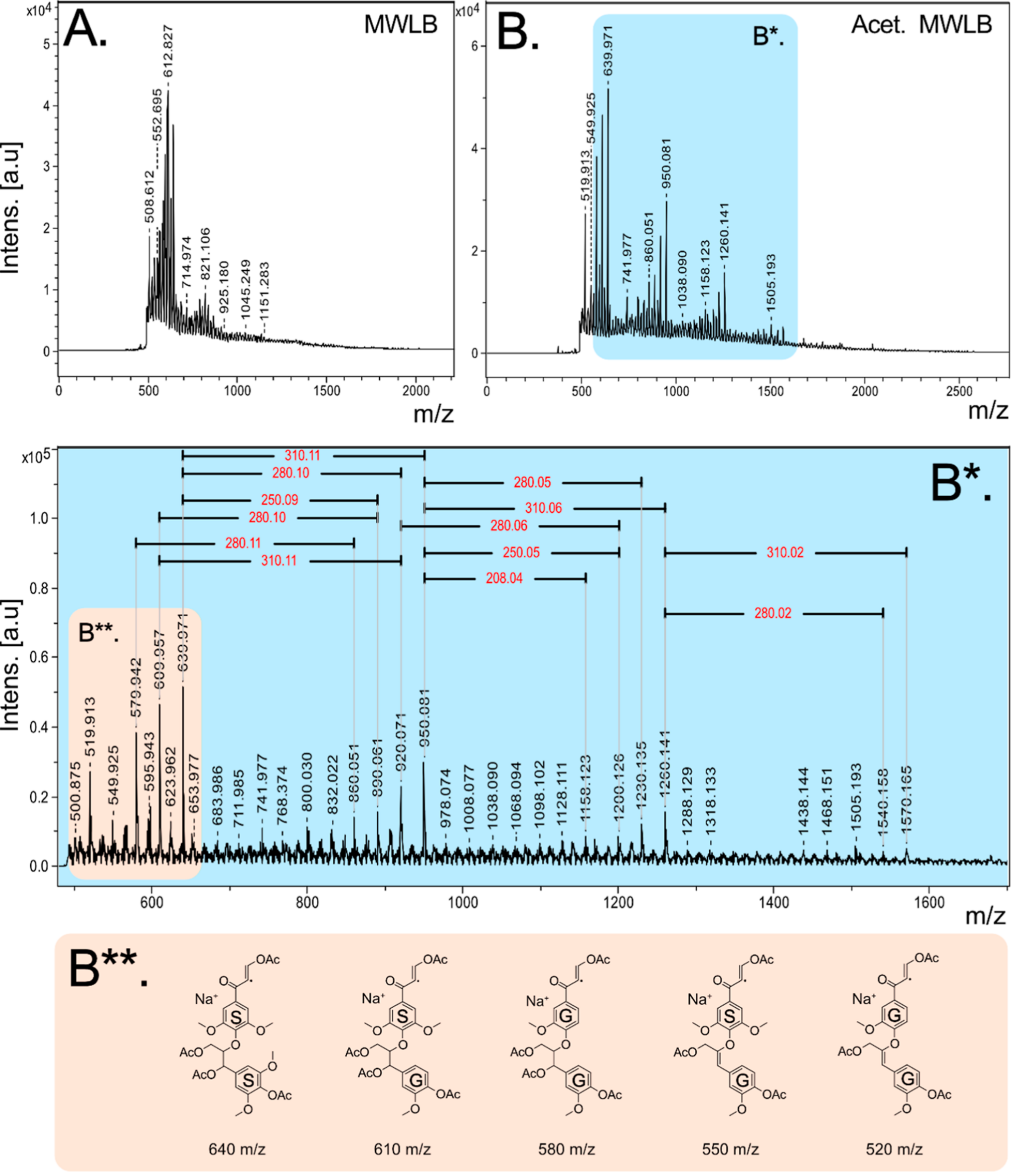
MALDI-TOF MS spectra of birch MWL (A), acetylated birch MWL (B,B*), and tentative structures for starting dimers (B**), interpreted as sodium adducts, from which polymerization patterns can be derived. All related spectra can be found in Figures S4 and S6–S9. Complete acetylation was confirmed by ^31^P NMR (Figure S3).

Having experimentally confirmed expected mass increments from theory, we now revert to constructing an LPM by focusing on the high-intensity peaks (a simplified statistical analysis for chosen oligomer peaks is provided in Table S1). In LPM construction, we start with a low-mass adduct, used as a molecular anchor, and then progress through the clusters, identifying the mass increments by the principle of endwise addition of monomers into the chain. For the complete structure, the exact molecular structure of the starting low-molecular-weight adduct is required. In our earlier work, we identified a low-MW adduct as an aromatic structure with an enone or enal structure on the aliphatic side chain.^22^ It was suggested that this structure contains a stabilized radical, either already present in the MWL sample or as the result of a MALDI event. These structures, we believe, are consistent with homolytic cleavage reactions that occur during the ball-milling used to isolate the lignin. Similarly, in the present study, HSQC and HMBC analyses have provided evidence of both enal and enone structures in Birch MWL. From the MALDI-TOF MS spectra, we identified adduct peaks at 520, 550, 580, 610, and 640 *m*/*z* (Figure 5B**) and proposed these to correlate to candidate structures with enone configurations containing a radical. The radical may be present within the sample or as a result of MALDI events. Furthermore, these are interpreted as sodium adducts, as these gave the most plausible structures for the given *m*/*z*. Sodium is a ubiquitous impurity present almost everywhere, such as in glassware and solvents. In previous MALDI-TOF MS analysis on lignin model dimer compounds, the acetylated lignin dimers were detected as adducts with sodium and potassium, though no additional cationization agents were supplied.^10^ Building from these assumptions and the resulting structures, LPMs of some selected oligomers were derived (Figure 6).

**Figure 6:**
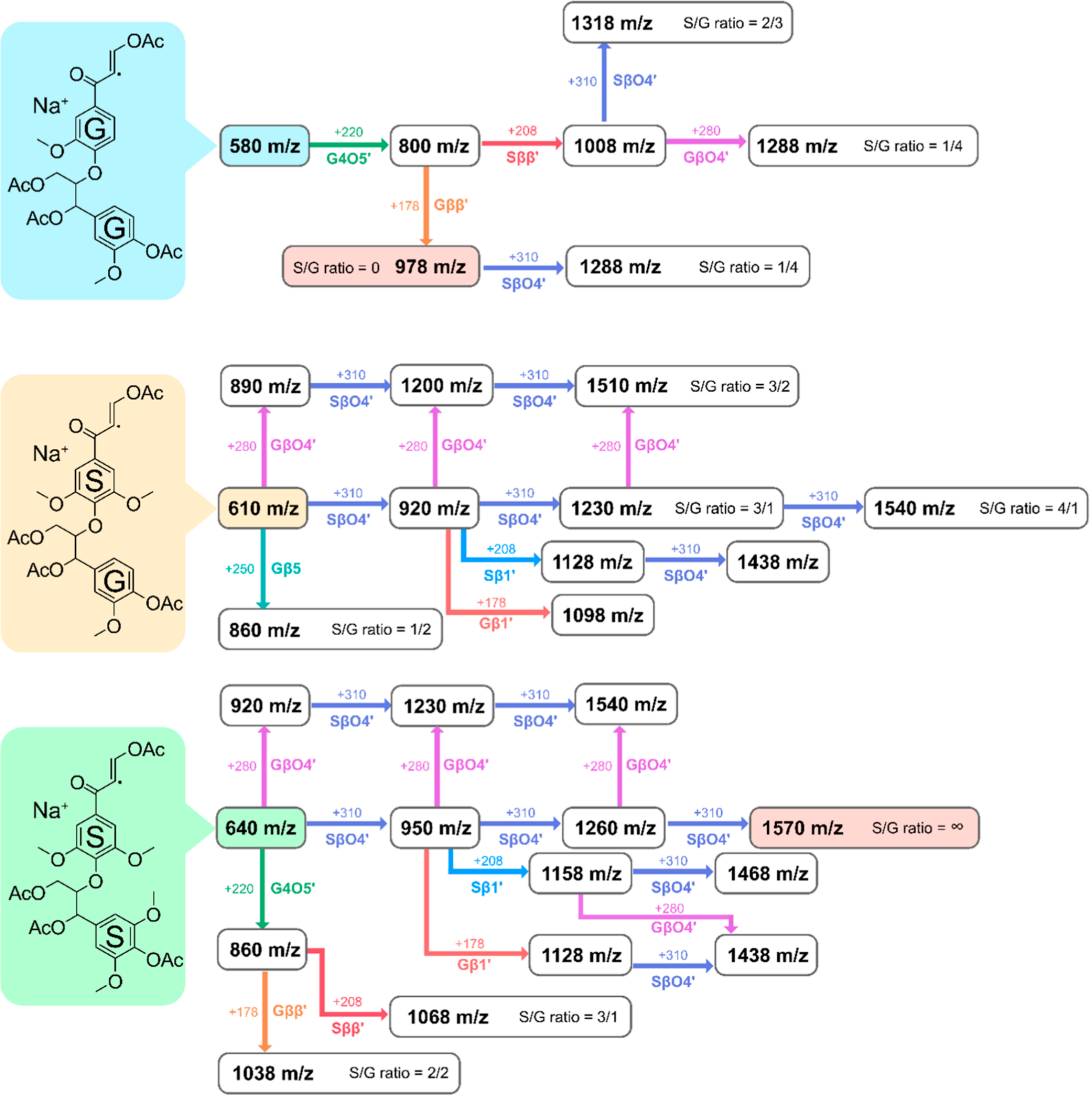
Linkage progression map for oligomers in Birch MWL. 310 represents the addition of an SβO4′, 280 represents the addition of a GβO4′, 250 represents the addition of S4O5′ or Sβ5′, 220 represents the addition of either a G4O5′ or Gβ5′, 208 represents the addition of either an Sββ′ or a Sβ1′, and 178 represents the addition of a Gββ′ or a Gβ1′. Three adducts at 580, 610, and 640 *m*/*z* were used as starting molecular anchors. The structure of 640 was confirmed with MALDI-TOF MS^2^ (Figures 7, S13, and S14).

Earlier, it was shown that although acetylation achieved a partial discernment of condensed bonds, it was not able to discern between β1′ and ββ′ or β5′ and 4-*O*-5′ due to similar mass increments when these bonds are formed. Interestingly, though, we provide further insights on the full discernment of these bonds based on the LPM iteration together with the known principles of lignin polymerization. More specifically, if an increment of 178 or 208 is end-wise added, this could only imply an addition of β1 and not ββ′. This is due to the fact that a ββ′ cannot form from a phenolic end of the growing polymer. The only way to form a ββ′by endwise addition to the growing polymer is to change the direction of the polymerization, which would require that a 4-*O*-5′ bond be formed first. Thus, a mass increment of 220 followed by a 178 increment implies a progression 4-*O*-5′ followed by ββ’. Note that the 220 in this case cannot be a β5′. With this added knowledge, where β1′, ββ′, β5′, and 4-*O*-5′ are discerned, LPMs are constructed in a more structurally precise manner. Selected oligomer peaks derived from the LPM framework have been further validated by MS^2^, which will be discussed in a later section. From the LPM (Figure 6), we can start to classify oligomers. For instance, we observe some LPMs leading to homo-oligomers consisting only of aryl ether bonds. In this category, 15 adducts from the most intense adduct peaks in the spectra, consisting of dimers to pentamers, are identified in the LPM. The aryl ether homo-oligomers detected may contain only S-units, e.g., 1570 *m*/*z* with an infinite S/G ratio, or predominantly S-units, e.g., 1540, 1510 *m*/*z*, with S/G ratios 4:1 and 3:2, respectively.

Apart from aryl ether-only containing oligomers, 13 adducts with mixed condensed and noncondensed interunit linkages are reported (Figure 6). These stem from the same base dimeric structures used to construct the aryl ether-only oligomers, i.e., 580 *m*/*z*, 610 *m*/*z,* and 640 *m*/*z*. However, the signal intensities related to these proposed nonaryl ethers containing structures such as ββ′, β5′, and 4O5′ are weak but still statistically valid (Table S1) and consistent with results from the HSQC analysis, at least for ββ′ and β5′. Interestingly, unlike the case of softwood MWLs, 4-*O*-5′ structures escape analysis by HSQC due to the presence of overlapping S 2, 6 signals (Figure 2). The MALDI-TOF MS analysis, however, can complement their analysis. Following the LPM derived from *m*/*z* 580 in this context, we observe that a G4O5′ is followed by a Gββ′ linkage. Similar observations were made in our previous study on Spruce MWL, albeit with a G-based ββ′ linkage presiding next to a G-based 4O5′. This observation is interesting since ββ′ has always been closely associated with βO4′. In fact, ββ′ is believed to form as dimers. Here, however, the presence of a 4O5′ next to ββ′ suggests alternative routes for incorporation of a ββ′ linkage in the growing polymer. More specifically, a 4O5′ linkage formed by the coupling of a phenoxy radical on the polymer to the C5 radical of the incoming monomer will have possibilities to incorporate a ββ′ linkage. It is also important to note, as earlier discussed, that β1′ formation can be distinguished from ββ′. Nevertheless, for the oligomers consisting of mixed interunits, e.g., 860, 1038, 1288, and 1318 *m*/*z*, the S/G ratios are observed to be lower than or equal to 1. The observations on the molecule-specific S/G ratios provide new perspectives on a few things;

1 They suggest that the supply of the monolignol type to the cell wall is somehow regulated such that sometimes only sinapyl alcohol is supplied, other times only coniferyl alcohol, and sometimes both. This may be necessary to fulfill cell-type-specific function based on structure–property relationships.
2 The global S/G ratio does not reflect the actual ratio within individual molecules in a given sample. As seen here, there are S/G ratios that span from 0 (oligomers with only G units, Figure 6) to infinity (oligomers with only S units, Figure 6). For example, the HSQC results discussed earlier estimate an S/G ratio of 2.3, which is consistent with the literature for hardwoods^5,6,23,24^ and pyrolysis GC measurements.^25^ Furthermore, S units seem to be more expressed in aryl ether structures, while G-units appear to be predominant in the condensed-type interunits.

The LPM approach, achieved using MALDI-TOF MS, therefore, provides deeper insights into the lignin structure and heterogeneity. While the reasoning behind the theory for the framework appears logically sound, the structures proposed in the LPM need to be further evaluated. In peptide characterization, de novo sequencing utilizes MS^2^ in MALDI-TOF MS analysis of peptides to elucidate the structures due to the specific fragmentation patterns that yield the product ion spectra. The product ion spectra can be interpreted either by hand or through matching against a database.^26–28^ Carrying out further MS^2^ studies on selected lignin oligomers should be able to either strengthen or disqualify the structures proposed in the current LPM and provide insight into whether there is potential to improve upon this work to achieve “true” de novo sequencing for lignins.

### MALDI-LIFT-TOF-TOF MS^2^

To further investigate the validity of the LPM, adducts 640, 860, 890, 920, 950, 1158, 2100, 1230, 1260, and 1570 *m*/*z* were studied by MALDI-TOF MS^2^ (Figures 7, S5, and S10–S20). A comprehensive elucidation of the fragmentations is provided in Figure 8, where spectra for investigated adduct peaks at *m*/*z* 860, 1158, 1230, and 1570 are used as illustrative examples. All found fragments for each adduct MS^2^ spectra can be found in Figures S16–S20. Figures 7 and 8 are discussed simultaneously.

**Figure 7:**
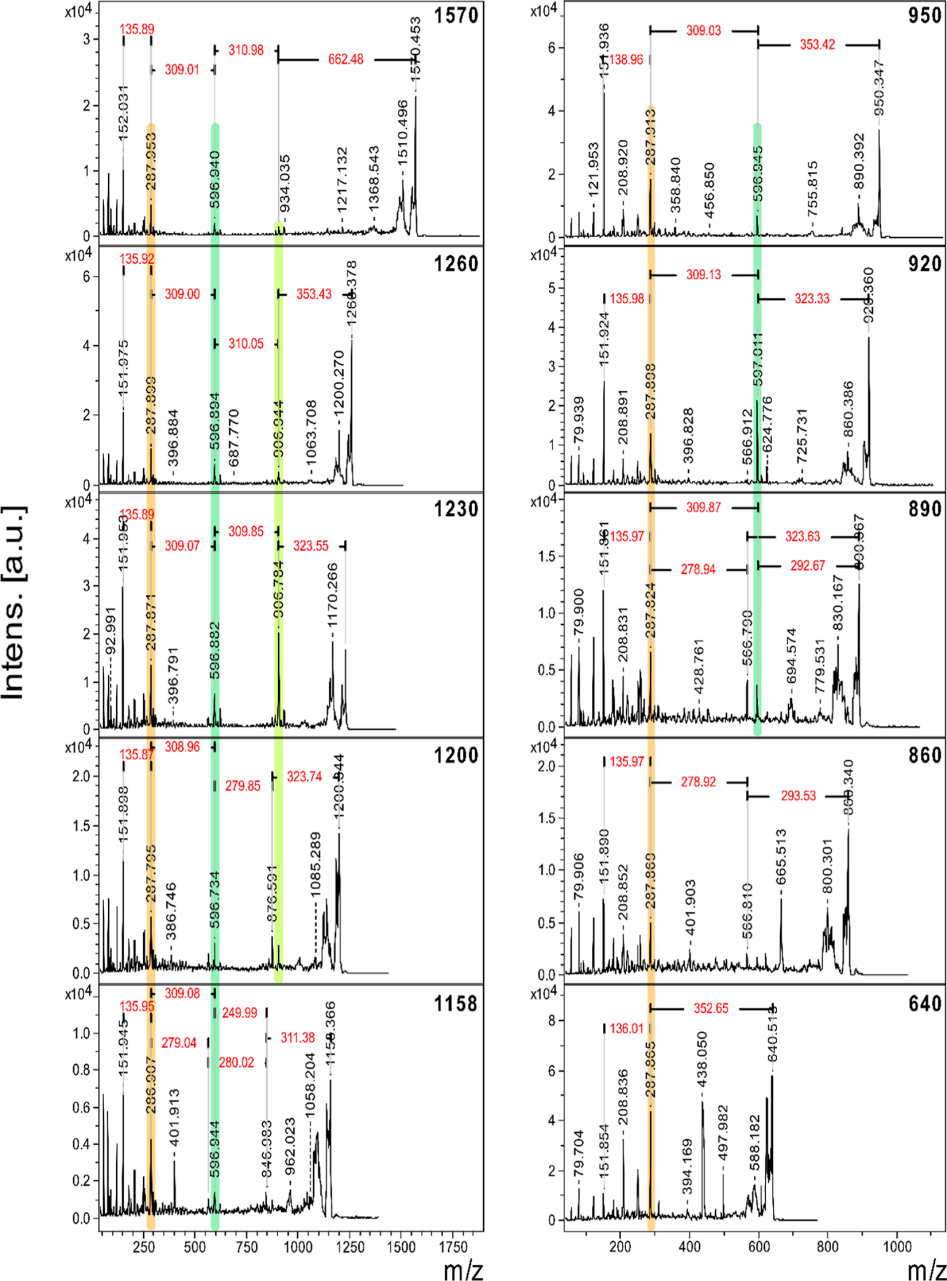
MALDI-LIFT-TOF-TOF MS^2^ spectra of adducts at (left) 1570, 1260, 1230, 1200, and 1158 and (right) 950, 920, 890, 860, and 640 *m*/*z*. Parent and fragment spectra are overlaid in the same spectra. Fragments of the same *m*/*z* within different adduct spectra are highlighted in the same color.

**Figure 8:**
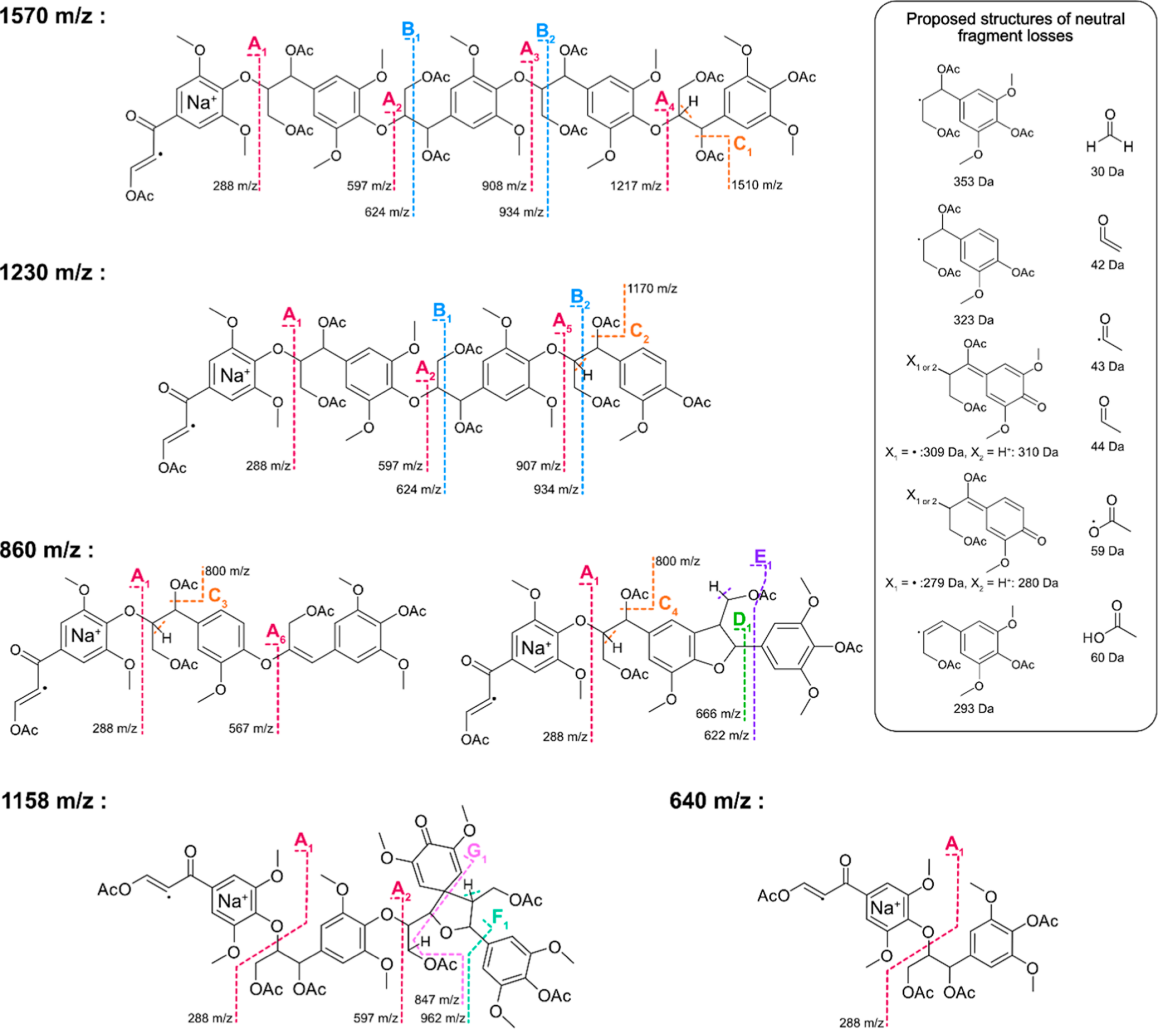
Tentative structures of investigated lignin oligomers in LID-induced MALDI-TOF/TOF MS^2^. Here, the fragmentation for tentative structures found at adduct peaks 640, 860, 1158, 1230, and 1570 *m*/*z* are used as illustrative examples. Proposed structures of neutral fragment losses are given (right) for some of the observed mass losses. All recreated fragment structures for all investigated adducts are given in Figures S16–S20.

It has been shown in previous literature that MS^*n*^ on sodiated lignin species suffer from little to no fragmentation, typically with poor signal.^17,29^ Evidently from our work, there is fragmentation and retention of positive charge of the acetylated lignin analytes in MALDI-TOF MS^2^ when utilizing LID. All investigated parent ions yield fragment peaks, and there seems to be highly specific fragmentation patterns reoccurring for different oligomers. In general, mass losses of 60 Da, likely corresponding to acetic acid (HOAc), are observed from the parent ion in MALDI-TOF MS^2^. This would either occur as a single step, where HOAc is lost as a result of π-bond formation, or as successive steps, where the loss of acetaldehyde (42 Da) is immediately followed by the rapid loss of water (18 Da). When analyzing the fragment region close to the parent ion, some mass differences could be interpreted as partial loss of the acetyl group (42, 43, and 44 Da, Figure S15). There seems to be a recurring fragmentation pattern where cleavage occurs at the βO4′ linkage, essentially giving the same mass difference as observed in MALDI-TOF MS. Cleavage at the phenolic site of lignin model oligomers in other types of MS has been observed.^8,13,15,17^ However, it should be noted that the mechanism behind the fragmentation could be different, and comparisons between various MS techniques should be made with caution. As a support for the cleavage reaction at the phenolic site in MALDI-TOF MS^2^, SβO4′ losses are found in MS^2^ as either 353 (end-unit) 309 or 310 (chain-unit) and GβO4′ can be found as either 323 (end-unit) 279 or 280 (chain-unit) Da in difference between fragment peaks. The most recurring fragment peaks found in most spectra are 258, 288, 567, 597, 877, and 907 *m*/*z*, and these all seem to stem from cleavage at the phenolic site. There is a recurring difference of 30 Da between several major fragment peaks. The reason for this specific difference is postulated to occur for either of two reasons: (1) some adduct peaks can contain both S- and G-units, and this showcases the possibility of S- and G-units being randomly distributed within the chain which causes fragment peaks to appear with 30 Da difference, or (2) there is a loss of formaldehyde (CH_2_O) during the fragmentation process.

The MALDI-TOF MS^2^ analysis revealed a shared and distinct molecular fragment at 597 *m*/*z* for all investigated oligomers, except for those at 640 and 860 *m*/*z* (Figure 7). From the 597 *m*/*z* dimeric structure, there is a mass difference of 309 to the fragment peak at 288 *m*/*z*. We propose that the 309 and 310 Da difference both correspond to an SβO4′ chain unit, but the fragmentation either retains or loses a hydrogen (Figure 8). A plausible structure for the 288 *m*/*z* fragment suggests that this is an S-unit containing either an enone or enal structure in the aliphatic side chain. For instance, the 1260 *m*/*z* adduct fragmentation pattern suggests a homolytic cleavage with loss of an acetylated S-monomer end-unit radical with a mass of 353 Da to give a fragment peak at 907 *m*/*z*. If fragmentation occurs at the aryl ether unit in the middle of the chain, this would yield the fragment peak at 597 *m*/*z*. By the same logic, if cleavage occurs at the aryl ether linkage closest to the aliphatic end unit, this yields a fragment peak at 288 *m*/*z*. By a similar mechanism, a loss of ∼353 from adduct 640 *m*/*z* yields the fragment peak at 288 *m*/*z*, since this is the only possible cleavage at a phenolic linkage between the aliphatic and phenolic end units.

Adduct *m*/*z* 950 would, according to regular MS analysis, be a trimer with at least one SβO4 bond (see MALDI-TOF MS spectra in Figure 5B* and LPM in Figure 6). This hypothesis is further strengthened by MS^2^ analysis on the acetylated sample, which shows fragmentations that yield losses of 353 (SβO4′ end unit) and 309 (SβO4′ chain-unit) between major fragment peaks from the parent adduct peak to the final monomeric fragment peak at 288 *m*/*z*. For the adduct at 920 *m*/*z*, the LPM (Figure 6) suggests the presence of one G-unit and two S-units, based on the connectivity of βO4′ linkages. In MS^2^, this is further strengthened by the loss of 323 (GβO4′ end-unit), followed by the loss of 309 (SβO4′ chain unit) to 288 *m*/*z*. The faint peak at 567 *m*/*z*, which is 279 *m*/*z* away from 288 *m*/*z*, indicates the possibility of an oligomer present with a GβO4′ chain unit and an SβO4′ end unit present. Nevertheless, the presence of one G-unit and two S-units within the 920 *m*/*z* adduct is confirmed, and the order of the linkages can be elucidated from the fragmentation pattern.

When examining the 890 *m*/*z* adduct peak, two distinct oligomers are revealed, comprising an altered aliphatic end in conjunction with an S unit and a G unit. Compared to the fragmentation for the 920 *m*/*z* adduct, the 567 and 597 *m*/*z* fragment peaks are relatively close in intensity. The S- and G-units can reside either in the middle of the chain or as an end group (if the S end unit is in the form of an enol ether) since both 310 and 279 *m*/*z*, as well as 324 and 293 *m*/*z* differences, and the respective peaks for the resulting fragments are observed.

The 860 *m*/*z* adduct in the LPM could consist of three G-units. This is clearly not the entire truth since the 288 *m*/*z* fragment adduct is present, which, according to our construct, corresponds to the modified S-based aliphatic end unit. There is an increment of 279 (GβO4′ chain unit) to the fragment peak at 567 *m*/*z*. The end unit increment between 567 *m*/*z* and 860 *m*/*z* is ∼293/294 (the loss is denoted as 293.53 *m*/*z* in the Figure 7 MALDI-TOF MS^2^ spectrum for oligomer 860). One should note that 250/249 (postulated β5 chain-unit increment) plus an acetyl group (∼43/44) becomes 293 Da, which could indicate that this increment may signify a β5′ end-unit. Furthermore, if homolytic cleavage occurs on the α-carbon in the β5′-linkage it leads to the loss of the aromatic ring in the Sβ5′ end unit. The resulting fragment containing the sodium, aliphatic S-unit, GβO4′ chain unit, and the retained aliphatic chain that makes up the Sβ5′ linkage would have a mass of 666 Da (Figures 8 and S17). Evidently, there is a strong fragment peak at 666 *m*/*z* in the MS^2^ spectra for adduct 860 (Figure 7, 860). The possibility of this being the fingerprint of homolytic cleavage of an acetylated β5′ linkage should not be ruled out. Moreover, the 860 *m*/*z* peak is 250 away from the 610 *m*/*z* adduct, which has been proposed to be a dimer consisting of the modified aliphatic S-unit and a GβO4′ phenolic end unit. Hence, if this fragmentation of acetylated β5′ is possible, the structure also has support in the increments found in regular MS. Given the additional fragmentation pattern and the occurrence of the 293 Da loss in adduct 890, which should not be able to contain a β5′ end unit following a SβO4′ due to the C5 being blocked by a methoxy group, there could also be an S-based acetylated enol ether end group in the adduct peak at 860 *m*/*z*. Due to the distinctly different fragment patterns, we suggest that the 860 *m*/*z* adduct houses two different oligomers, where one is purely βO4′-based and the other contains a β5′ phenolic end.

From the recreated tentative structures, the identification of a pure SβO4′ pentamer (1570 *m*/*z*, Figures 7, 8, and S19) in MS appears to be confirmed by the examination of the fragmentation pattern in MS^2^. More specifically, there is an increment of 693 Da, which should correspond to an SβO4′ dimer unit with the phenolic end acetylated to the fragment peak at 908 *m*/*z*. Fragment peaks are found at *m*/*z* 597and *m*/*z* 288, that is, with a difference of 310 and 309 Da, respectively. There is additionally a weak fragment peak at 1217 *m*/*z*. This fragment peak is 309 (SβO4′ chain unit) away from the fragment peak at 908 and 353 (SβO4′ end unit) away from the parent adduct peak at 1570 *m*/*z*, which further strengthens the hypothesis of there being a purely SβO4′-based oligomer. However, it should be noted that 258 and 567 *m*/*z* fragment peaks can be found in this spectrum. As discussed earlier, there may be another oligomer observed at 1570 *m*/*z*, or there could have been a loss of CH_2_O during the fragmentation process. The hypothesis is consistent with previous literature on several oligomers appearing at the same *m*/*z*.^8,9^

The 1158 *m*/*z* adduct was chosen to investigate an adduct peak that possibly would contain something other than purely βO4′-based units. As with the other investigated adducts, fragmentation may suggest the possibility of several oligomers detected at this specific *m*/*z*. The recurring losses from βO4′-based cleavages are observed (279, 280, and 309). However, a final loss of 311 suggests a spirodienone (β1′)-containing oligomer. This assignment is based on the proposed cleavage in the spirodienone moiety, resulting in fragments with masses correlating to those found in the MS^2^ spectrum (Figure S17), and is consistent with the associated tentative LPM increment.

Overall, the MALDI-TOF MS^*n*^ study reveals an in-depth study of oligomers present in Birch MWL. While we have largely identified the MS^2^ fragmentation pattern for acetylated aryl ether-based oligomers, the acetylated Sβ5′ and spirodienone require further studies to confirm this pattern by enriching the less common interunit-containing oligomers. This may be achieved by selective depolymerization approaches or model compound studies, followed by LID-induced MALDI-TOF MS^*n*^ analysis.

A short discussion of the results is in order. Here, we show that the traditional S/G ratios, in the regime of 2–3, often used to describe the composition of a hardwood lignin, do not therefore reflect that of individual molecules. As shown, the S/G ratios span between 0 (G-only oligomers) and infinite (S-only oligomers). Furthermore, since these oligomers are clip-offs of lignin macromolecules, as shown here, the presence of S-only and G-only oligomers may also suggest the existence of block copolymers defined by segments of S units intercepted by segments of G units. The exact mechanism by which this can arise during lignin polymerization requires further investigation. However, a plausible hypothesis is that the monolignol type supplied to the cell wall during lignin polymerization is regulated such that S-units and G-units are supplied at different times. Consequently, when only S units are supplied, only S lignin is formed. A similar logic applies to the supply of only the G-units. If the hypothesis holds, then the formation of S-only aryl ether oligomers is quite palatable since S units are blocked at both 3- and 5-positions of the aromatic ring by methoxy groups and therefore can form mostly aryl ethers. G units, on the other hand, offer several bonding type possibilities due to the free 5-position.

## Conclusion

The combination of NMR and MALDI-TOF MS^*n*^ proves to be a powerful tool for providing an atomistic understanding of singular oligomers in heterogeneous lignin sample mixtures. We can determine the structure of individual oligomers in the sample through linkage progression mapping of MALDI-TOF-MS-derived cluster analysis on acetylated lignins. This significant milestone opens the door to deeper studies of lignin heterogeneity, which to date remains poorly defined.

For instance, we demonstrate for the first time that purely syringyl (S)-based and purely guaiacyl (G)-based oligomers coexist in the birch MWL. Oligomers containing combinations of S and G units were also identified. Moreover, the investigated oligomers all share a chemically modified aliphatic end unit that contains enone or enal structures. A combination of HMBC and MS^2^ has been used to validate the structural features proposed from MALDI-TOF MS analysis.

To conclude, minimal sample preparation without prefractionation of lignin and MALDI-TOF MS, supported by NMR, is sufficient to reconstruct molecular motifs of low-molecular-weight oligomers. The methodology was applied to technical lignins to evaluate the true potential of this simple analytical framework.

## Supplementary Material



## Notes

2D HSQC NMR spectrum and 2D HMBC NMR spectrum of nonmodified milled wood lignin from Birch, ^31^P NMR spectra of nonmodified and acetylated milled wood lignin from Birch, MALDI-TOF MS spectra of matrices, nonmodified milled wood lignin from birch, and acetylated milled wood lignin from birch, MALDI-TOF MS^2^ spectra of lignin oligomers, postulated structures from precursor and fragments found in MALDI-TOF MS^2^ analysis, and simple statistical analysis of *m*/*z* and S/N for oligomers included in linkage progression maps (PDF)

## Abbreviations

HSQC: heteronuclear single quantum coherence
NMR: nuclear magnetic resonance
HMBC: heteronuclear multiple bond correlation
MALDI: matrix-assisted laser desorption/ionization
TOF: time-of-flight
MS: mass spectrometry
MS^*n*^: tandem mass spectrometry
MWL: milled wood lignin
S: sinapyl
G: guaiacyl
βO4: β-ether
β5: pinoresinol
ββ: phenylcoumaran
DBDO: dibenzodioxocin
