# Supplementary material for: Linkage Progression Mapping: Precision Structure Analysis of Individual Oligomers in Birch Milled Wood Lignin
Source: Biomacromolecules. 2026 Mar 30;27(4):2839–50. doi: 10.1021/acs.biomac.5c02761 (PMC13080992; doi:10.1021/acs.biomac.5c02761)
Supplement: Supplementary file 1 [file bm5c02761_si_001.pdf]

Supporting information

Linkage Progression Mapping: Precision Structure Analysis of Individual Oligomers in Birch Milled Wood Lignin

Filippa Ludvig[ab], Åsa Emmer[c], Martin Lawoko[*ab]

[a] Wallenberg Wood Science Centre, Royal Institute of Technology, KTH, 100 44, Stockholm, Sweden

[b] Division of Wood Chemistry and Pulp Technology, Department of Fibre and Polymer Technology, School of Engineering Sciences in Chemistry, Biotechnology and Health, KTH Royal Institute of Technology, 100 44 Stockholm, Sweden

[c] Analytical Chemistry, Applied Physical Chemistry, Department of Chemistry, School of Engineering Sciences in Chemistry, Biotechnology and Health, KTH Royal Institute of Technology, 100 44 Stockholm, Sweden

Table of Contents

1. 2D and 1D NMR spectra 4

Figure 1. ^{1}H ^{13}C 2D-HSQC NMR spectra of non-modified milled wood lignin from Birch. 4

Figure 2. ^{1}H ^{13}C 2D HMBC NMR spectra of non-modified milled wood lignin from Birch. 5

Figure 3. ^{31}P NMR on acetylated milled wood lignin from Birch (2) and non-modified milled wood lignin from Birch (1). 6

2. MALDI-TOF MS and MS^2 Spectra 7

Figure 4. MALDI-TOF MS matrix spectra of 2,5-DHB 20 mg ml^{-1} crystallized from TA50. Region 0-5000 m/z has been investigated using positive mode with reflectron. Y-axis displays signal intensity (arbitrary unit) and x-axis displays mass-over-charge (m/z). 7

Figure 5. MALDI-TOF MS matrix spectra of saturated HCCA crystallized from TA50. Region 0-5000 m/z has been investigated using positive mode with reflectron. Y-axis displays signal intensity (arbitrary unit) and x-axis displays mass-over-charge (m/z). 8

Figure 6. MALDI-TOF MS spectra of non-modified milled wood lignin from Birch using 20 mg ml^{-1} 2,5-DHB in TA50 as matrix. Region 0-5000 m/z has been investigated using positive mode with reflectron. Y-axis displays signal intensity (arbitrary unit) and x-axis displays mass-over-charge (m/z). 9

Figure 7. MALDI-TOF MS spectra of acetylated milled wood lignin from Birch using 20 mg ml^{-1} 2,5-DHB in TA50 as matrix. Region 0-5000 m/z has been investigated using positive mode with reflectron. Sample was dissolved as 2.4 mg ml^{-1} (2 mg ml^{-1} in spectra title is incorrectly rounded off, lab notes dictate 2.4 mg ml^{-1}) in DMF. S-M application. Y-axis displays signal intensity (arbitrary unit) and x-axis displays mass-over-charge (m/z). 10

Figure 8. Expansion of 500-1700 m/z region of MALDI-TOF MS spectra of acetylated milled wood lignin from Birch using 20 mg ml^{-1} 2,5-DHB in TA50 as matrix**.** Sample was dissolved as 2.4 mg ml^{-1} (2 mg ml^{-1} in spectra title is incorrectly rounded off, lab notes dictate 2.4 mg ml^{-1}) in DMF. Y-axis displays signal intensity (arbitrary unit) and x-axis displays mass-over-charge (m/z). 11

Figure 9. Expansion of 500-1400 m/z region MALDI-TOF MS spectra of non-modified milled wood lignin from Birch. Y-axis displays signal intensity (arbitrary unit) and x-axis displays mass-over-charge (m/z). 12

Figure 10. MALDI-TOF MS spectra of acetylated milled wood lignin from Birch using saturated HCCA in TA50 as matrix**.** Region 0-5000 m/z has been investigated using positive mode with reflectron. Y-axis displays signal intensity (arbitrary unit) and x-axis displays mass-over-charge (m/z). 13

Figure 11. Expansion of 450-1600 m/z region of MALDI-TOF MS spectra on acetylated milled wood lignin from Birch using saturated HCCA in TA50 as matrix. Y-axis displays signal intensity (arbitrary unit) and x-axis displays mass-over-charge (m/z). 14

Figure 12. MALDI-LIFT-TOF/TOF spectra of adducts 1570, 1260, 1230, 1200, and 1158 m/z in acetylated milled wood lignin from Birch using saturated HCCA in TA50 as matrix. Y-axis displays signal intensity (arbitrary unit) and x-axis displays mass-over-charge (m/z). 15

Figure 13. MALDI-LIFT-TOF/TOF spectra of adducts 950, 920, 890, 860, and 640 m/z in acetylated milled wood lignin from Birch using saturated HCCA in TA50 as matrix. Y-axis displays signal intensity (arbitrary unit) and x-axis displays mass-over-charge (m/z). 16

Figure 14. MALDI-LIFT-TOF/TOF spectra of adduct 640 m/z in milled wood lignin from Birch, compared to only parent and parent together with fragment spectra collected for pure HCCA matrix at matrix adduct found closest to 640 m/z. 17

Figure 15. Expansion of MALDI-LIFT-TOF/TOF spectra of adducts 950, 920, 890, 860, and 640 m/z in acetylated milled wood lignin from Birch with additionally marked increments. Y-axis displays signal intensity (arbitrary unit) and x-axis displays mass-over-charge (m/z)................. 18

3. Postulated structures derived from MS^2 19

Figure 16. Postulated parent and fragment structures from MALDI-LIFT-TOF/TOF spectra of 640 m/z. 19

Figure 17. Postulated parent and fragment structures from MALDI-LIFT-TOF/TOF spectra of 1158, 950, and 860 m/z. 20

Figure 18. Postulated parent and fragment structures from MALDI-LIFT-TOF/TOF spectra of 1230 and 1200 m/z. 21

Figure 19. Postulated parent and fragment structures from MALDI-LIFT-TOF/TOF spectra of 1570 and 1260 m/z. 22

Figure 20. Postulated parent and fragment structures from MALDI-LIFT-TOF/TOF spectra of 920 and 890 m/z. 23

4. Statistical analysis 24

Table 1. Statistical analysis of peaks used to create the linkage progression maps**.** 24

1. 2D and 1D NMR spectra

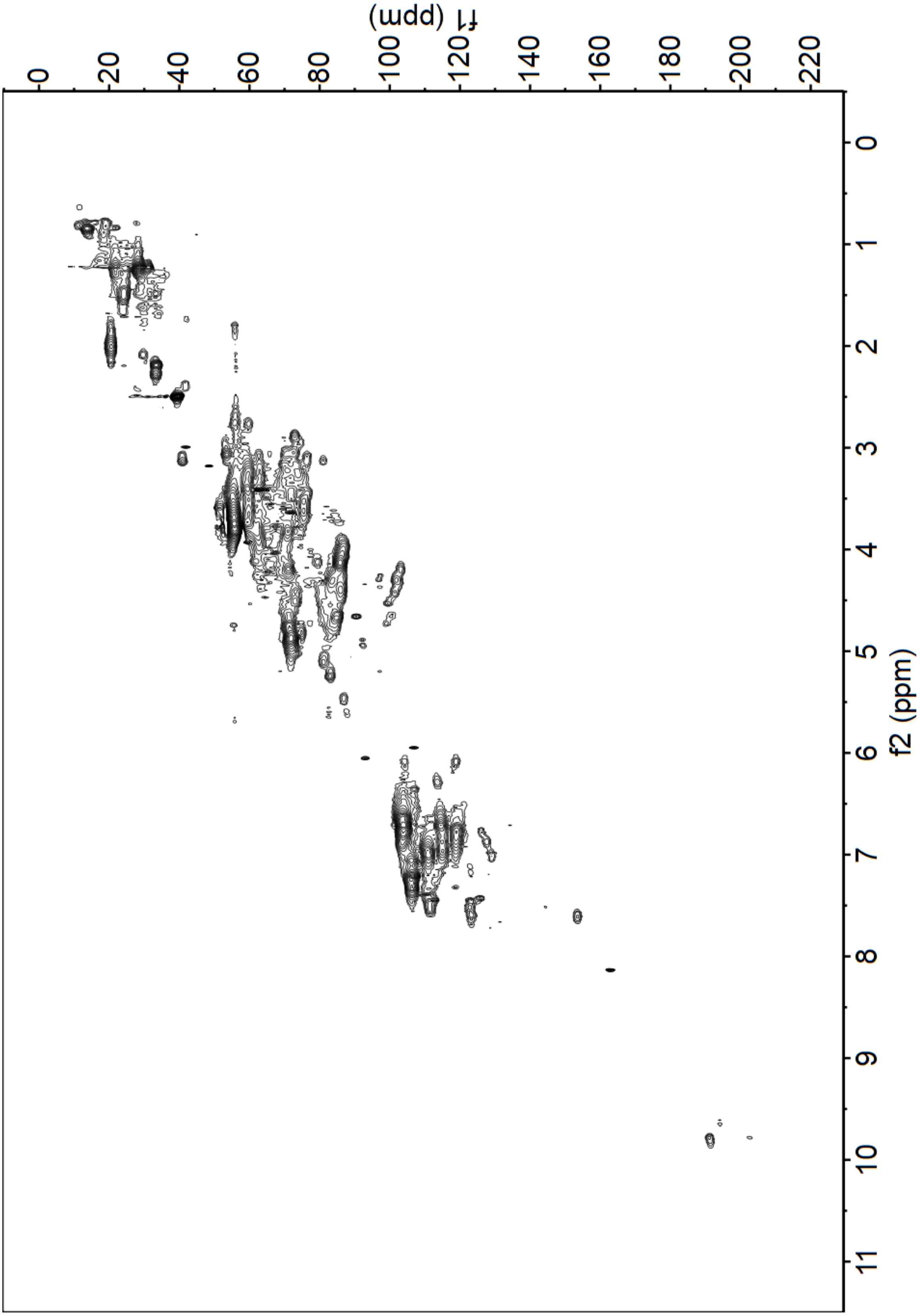

Figure 1. ^{1}H ^{13}C 2D-HSQC NMR spectra of non-modified milled wood lignin from Birch.

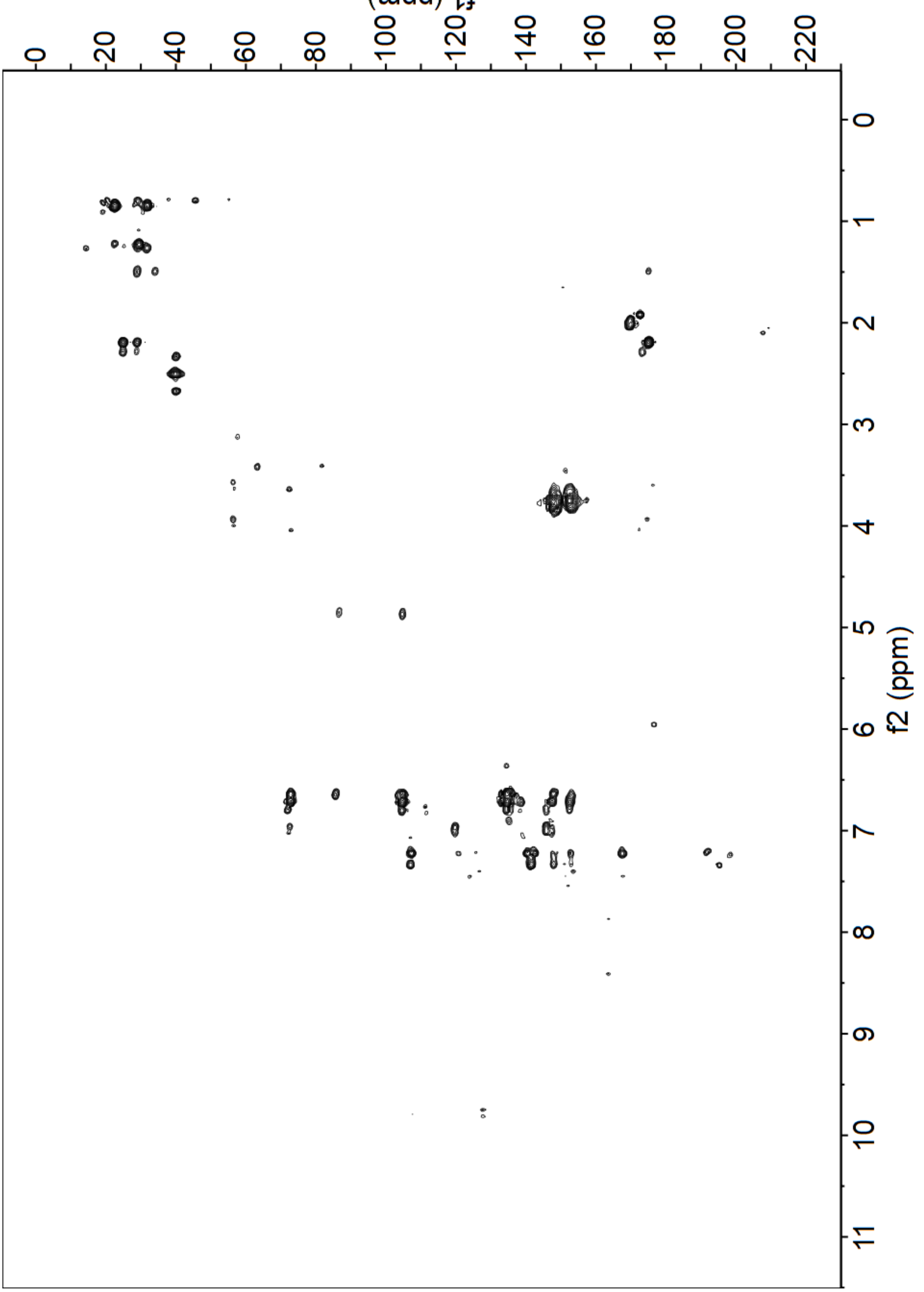

Figure 2. ^{1}H ^{13}C 2D HMBC NMR spectra of non-modified milled wood lignin from Birch.

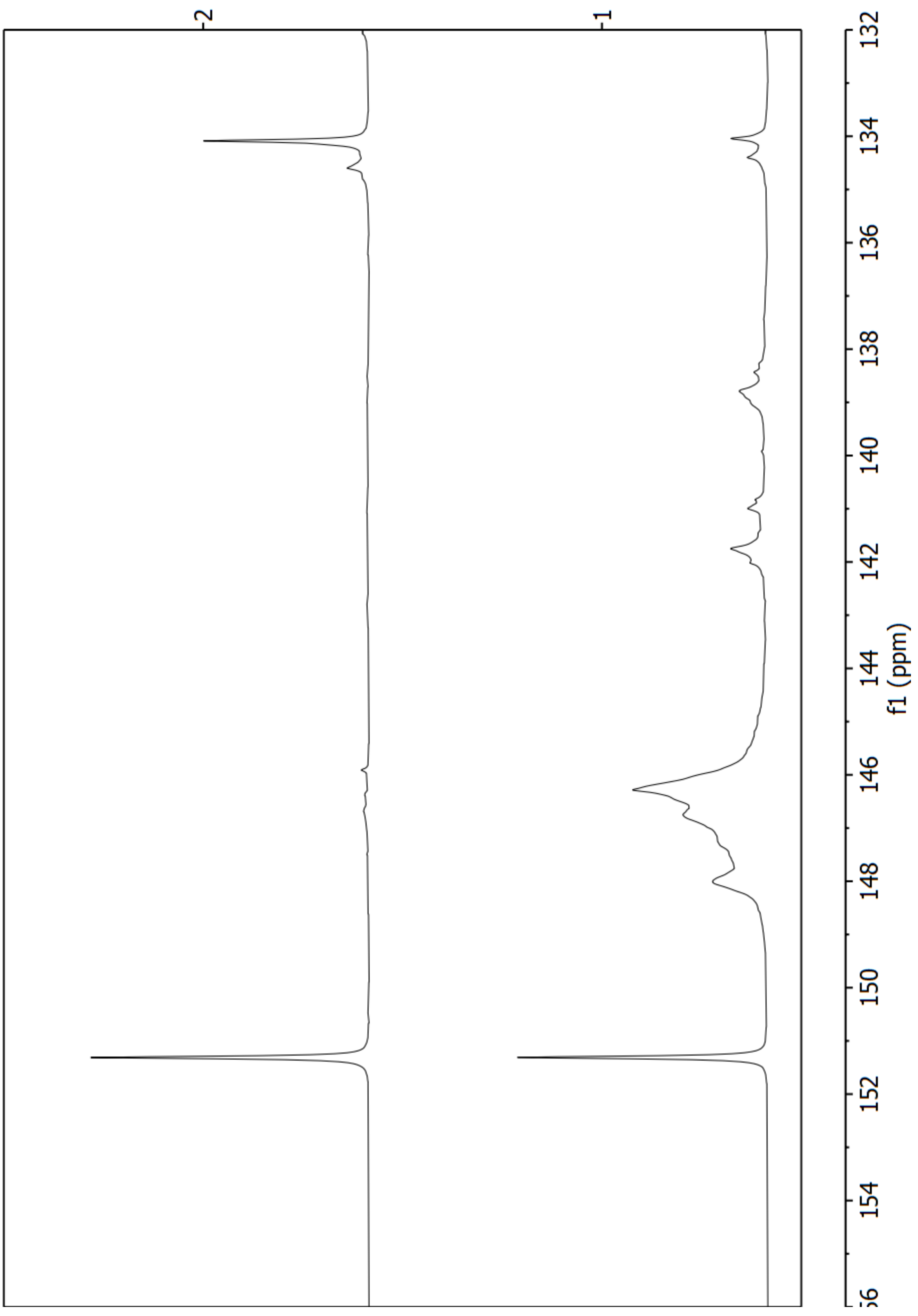

Figure 3. ^{31}P NMR on acetylated milled wood lignin from Birch (2) and non-modified milled wood lignin from Birch (1).

2. MALDI-TOF MS and MS^2 Spectra

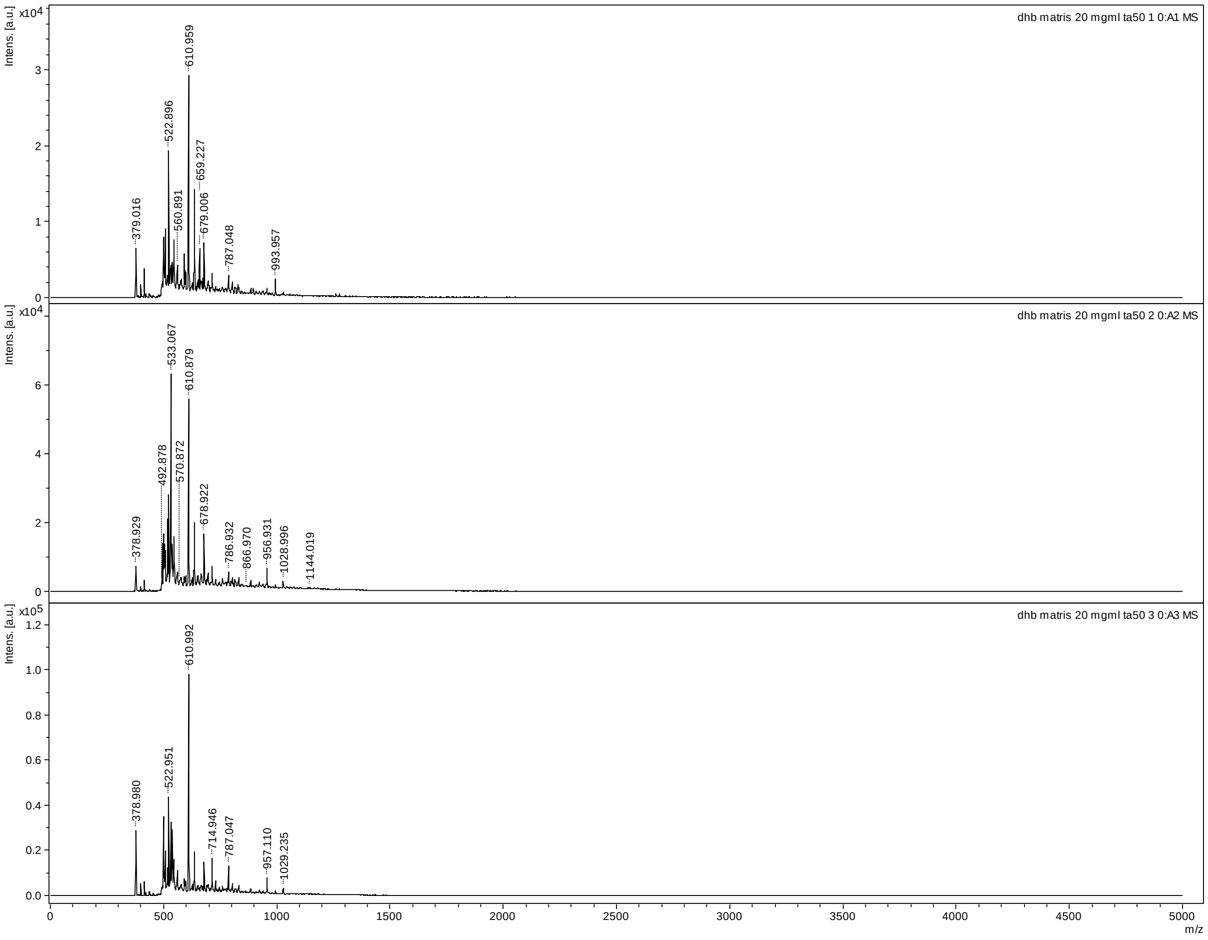

Figure 4. MALDI-TOF MS matrix spectra of 2,5-DHB 20 mg ml^{-1} crystallized from TA50. Region 0-5000 m/z has been investigated using positive mode with reflectron. Y-axis displays signal intensity (arbitrary unit) and x-axis displays mass-over-charge (m/z).

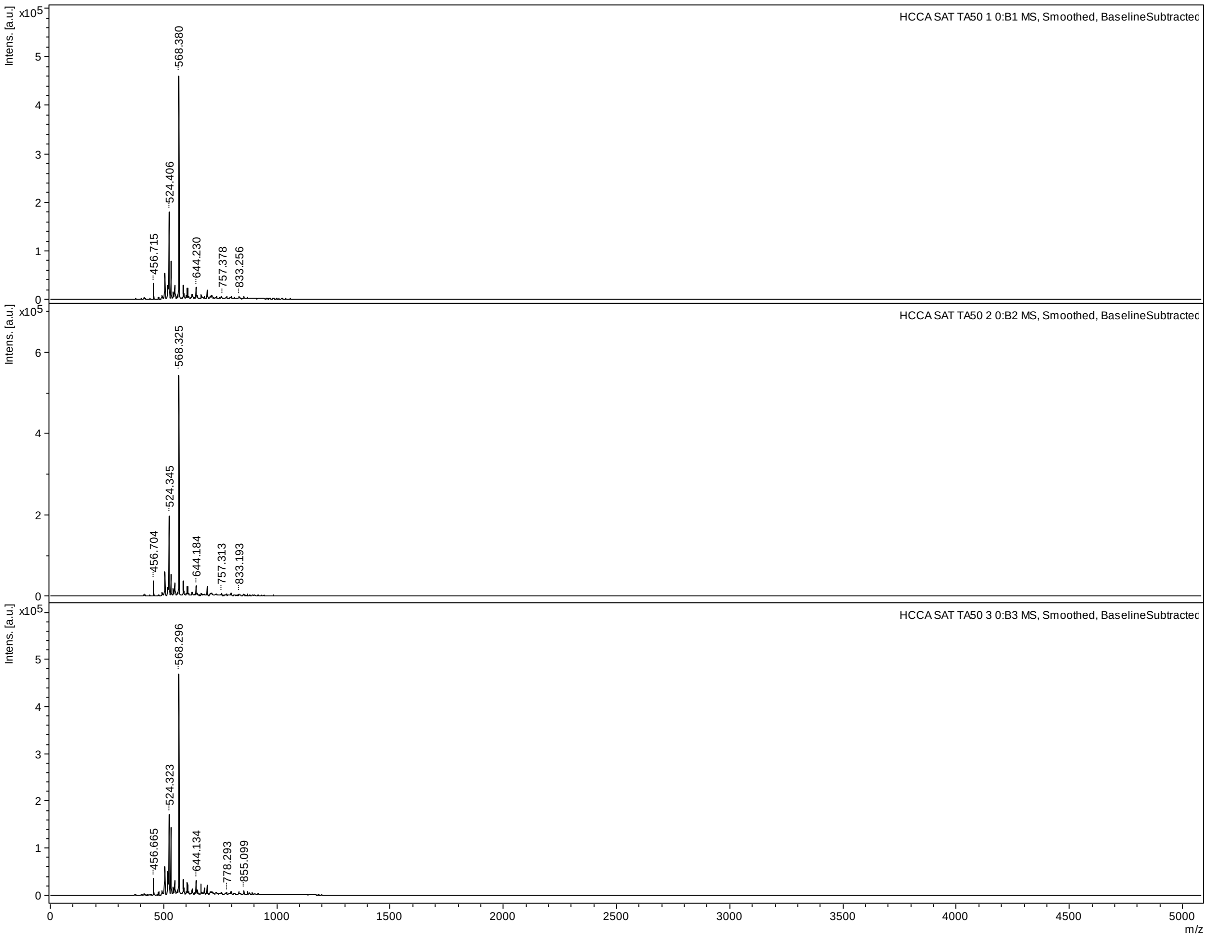

Figure 5. MALDI-TOF MS matrix spectra of saturated HCCA crystallized from TA50. Region 0-5000 m/z has been investigated using positive mode with reflectron. Y-axis displays signal intensity (arbitrary unit) and x-axis displays mass-over-charge (m/z).

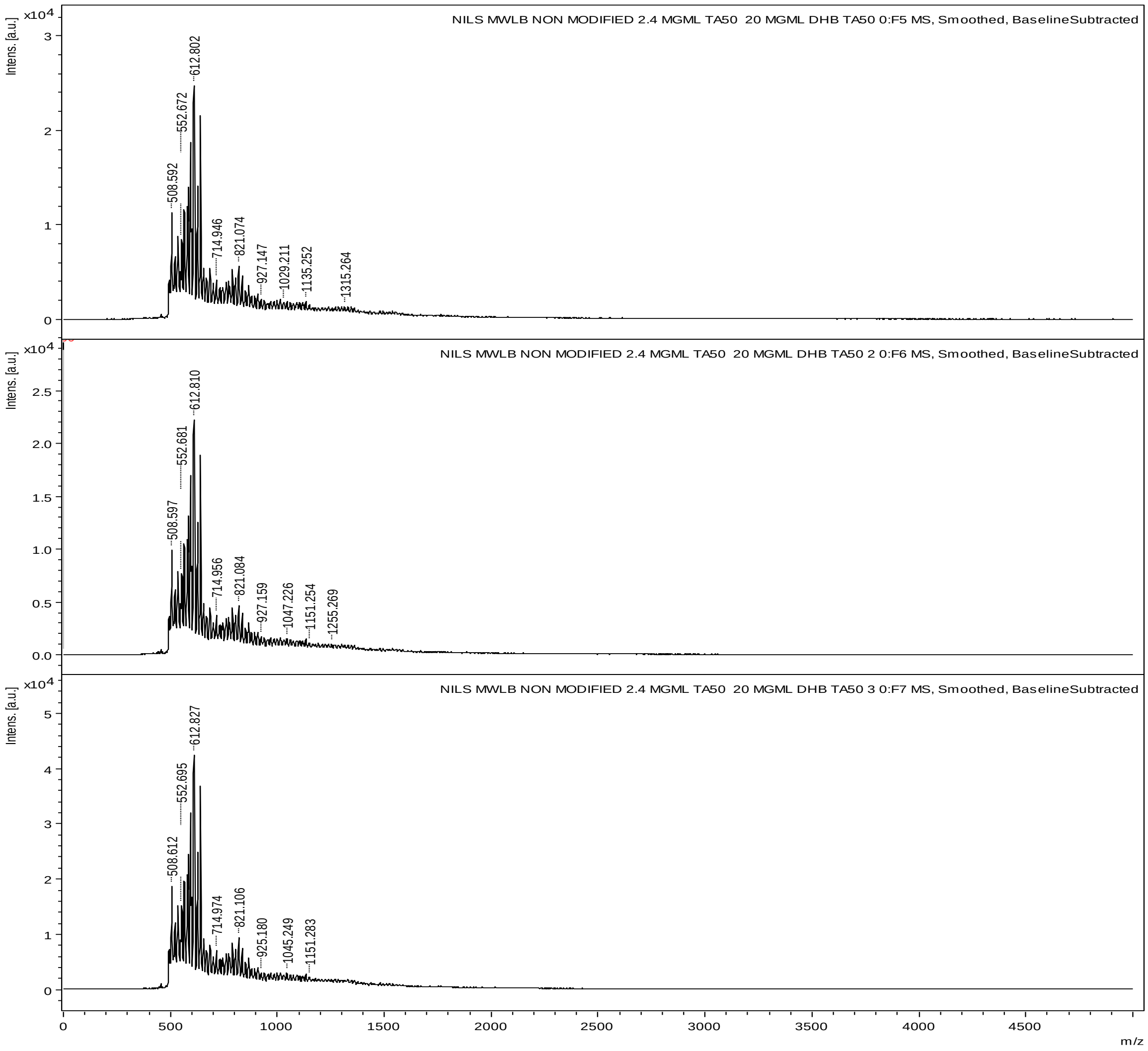

Figure 6. MALDI-TOF MS spectra of non-modified milled wood lignin from Birch using 20 mg ml^{-1} 2,5-DHB in TA50 as matrix. Region 0-5000 m/z has been investigated using positive mode with reflectron. Y-axis displays signal intensity (arbitrary unit) and x-axis displays mass-over-charge (m/z).

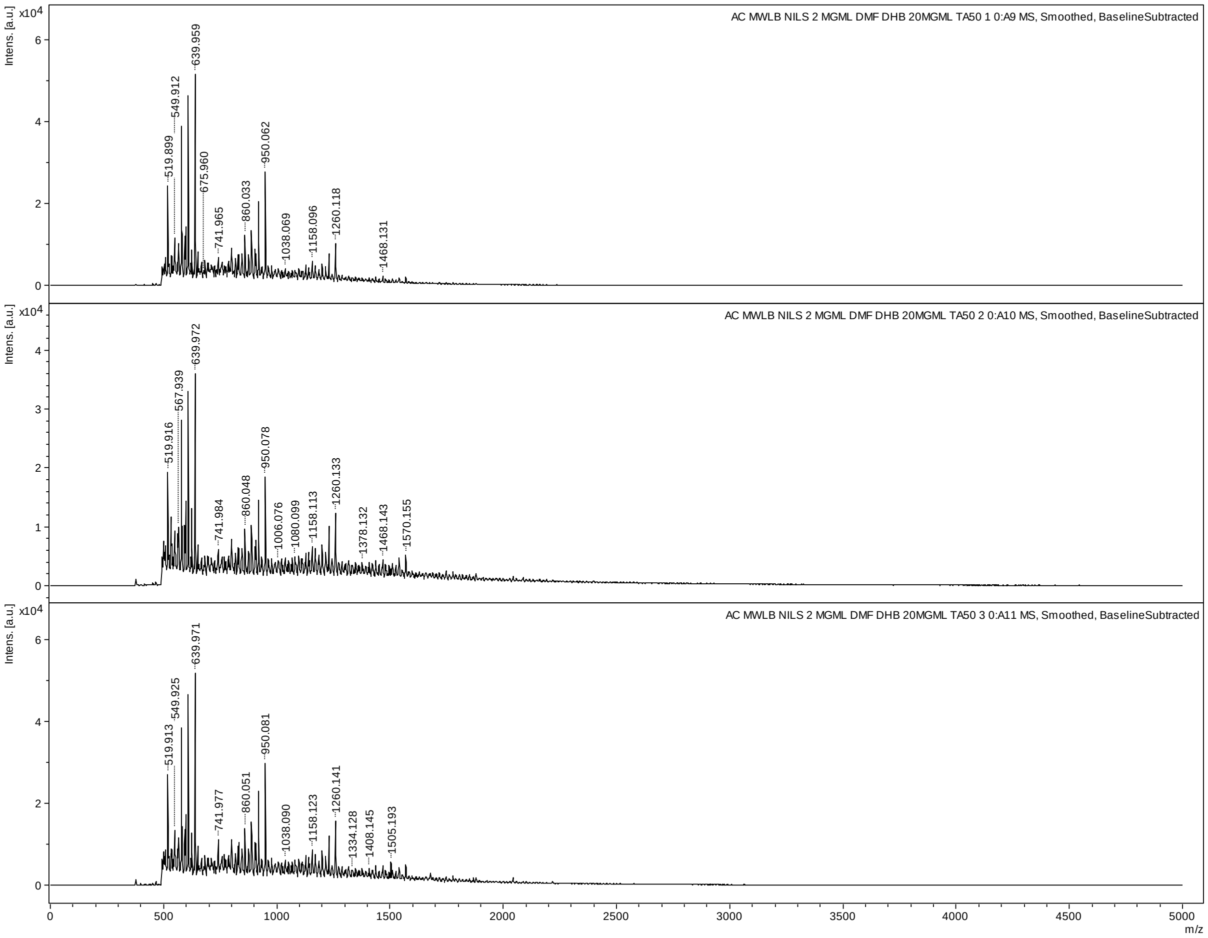

Figure 7. MALDI-TOF MS spectra of acetylated milled wood lignin from Birch using 20 mg ml^{-1} 2,5-DHB in TA50 as matrix. Region 0-5000 m/z has been investigated using positive mode with reflectron. Sample was dissolved as 2.4 mg ml^{-1} (2 mg ml^{-1} in spectra title is incorrectly rounded off, lab notes dictate 2.4 mg ml^{-1}) in DMF. S-M application. Y-axis displays signal intensity (arbitrary unit) and x-axis displays mass-over-charge (m/z).

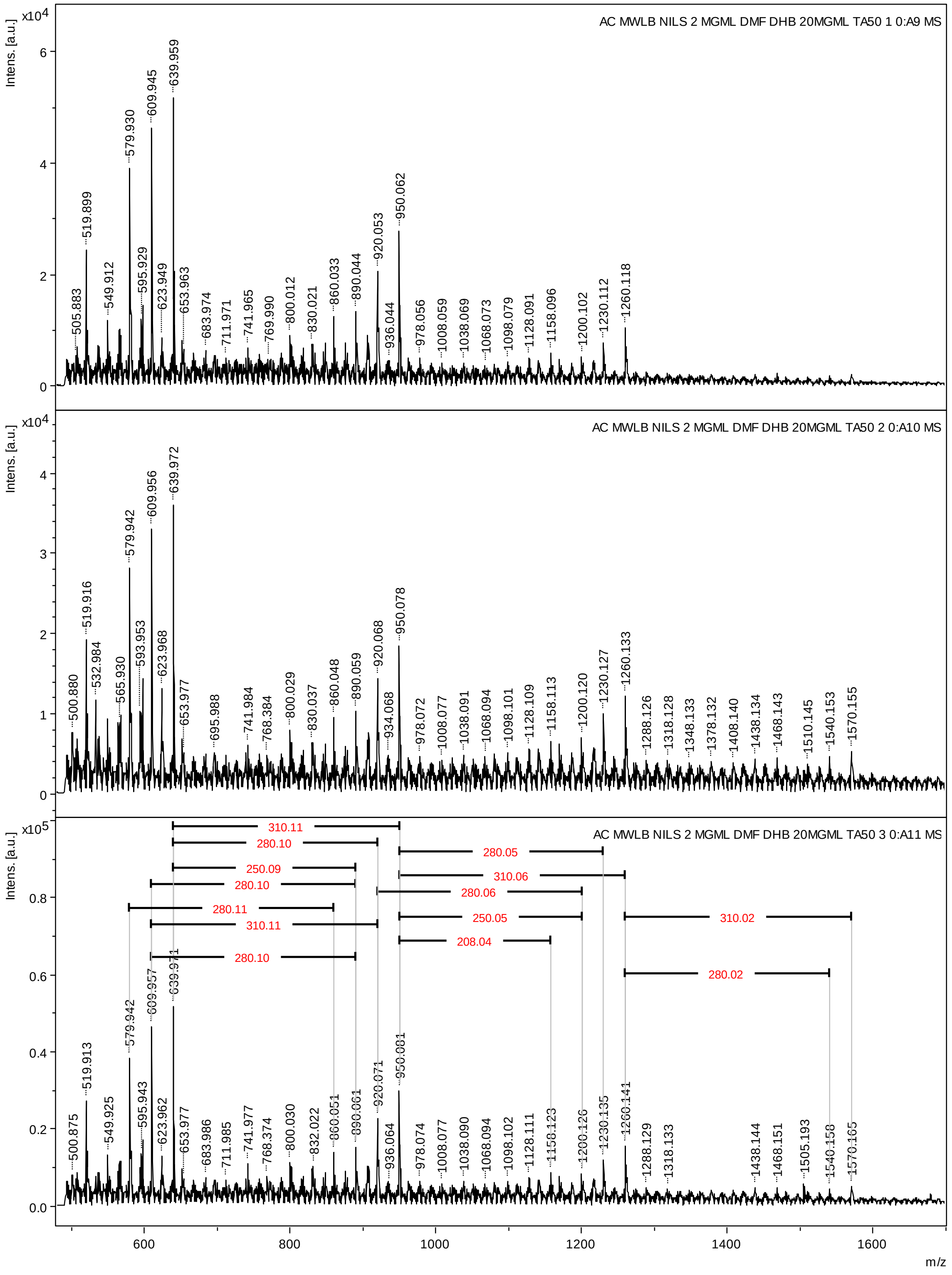

Figure 8. Expansion of 500-1700 m/z region of MALDI-TOF MS spectra of acetylated milled wood lignin from Birch using 20 mg ml^{-1} 2,5-DHB in TA50 as matrix**.** Sample was dissolved as 2.4 mg ml^{-1} (2 mg ml^{-1} in spectra title is incorrectly rounded off, lab notes dictate 2.4 mg ml^{-1}) in DMF. Y-axis displays signal intensity (arbitrary unit) and x-axis displays mass-over-charge (m/z).

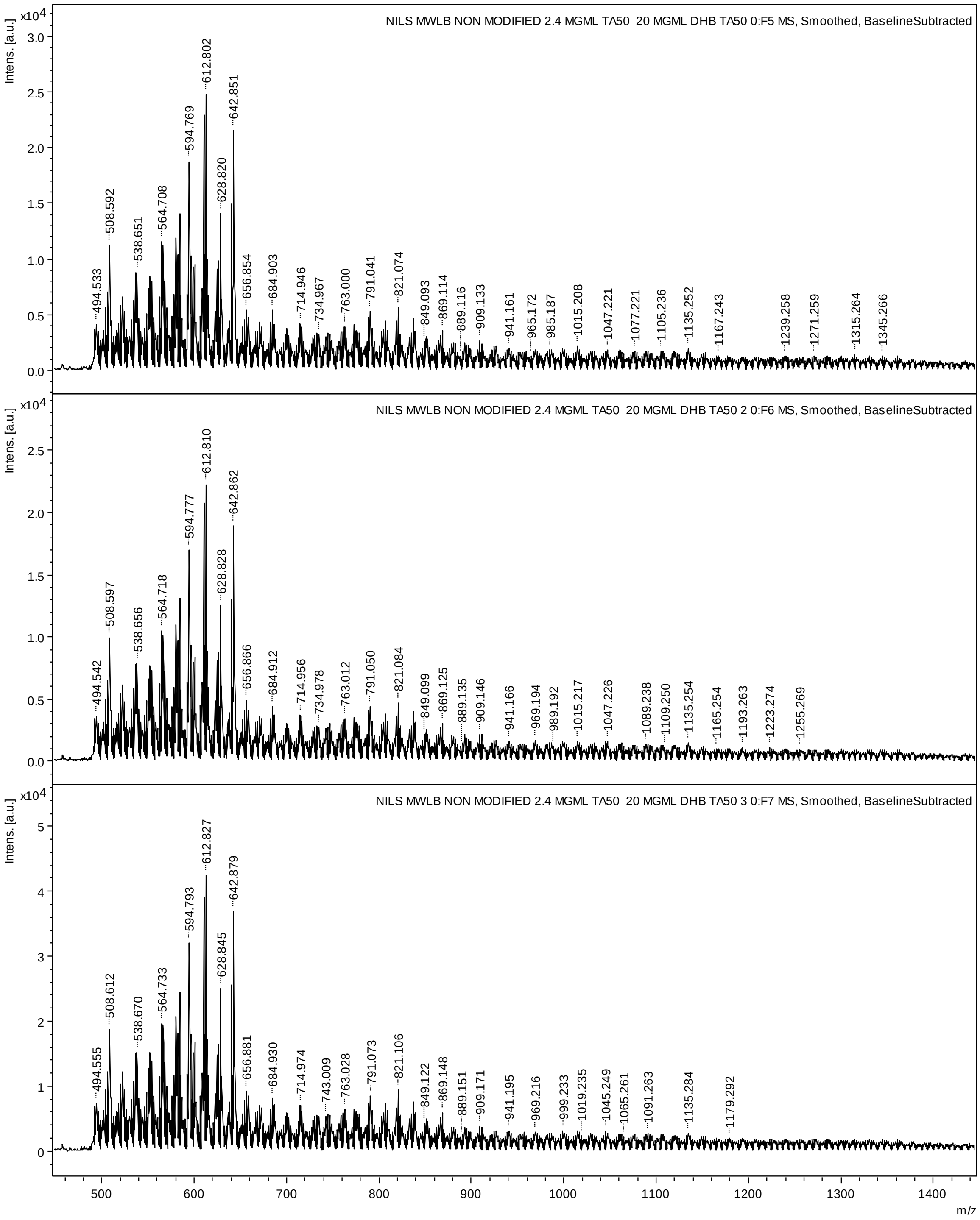

Figure 9. Expansion of 500-1400 m/z region MALDI-TOF MS spectra of non-modified milled wood lignin from Birch. Y-axis displays signal intensity (arbitrary unit) and x-axis displays mass-over-charge (m/z).

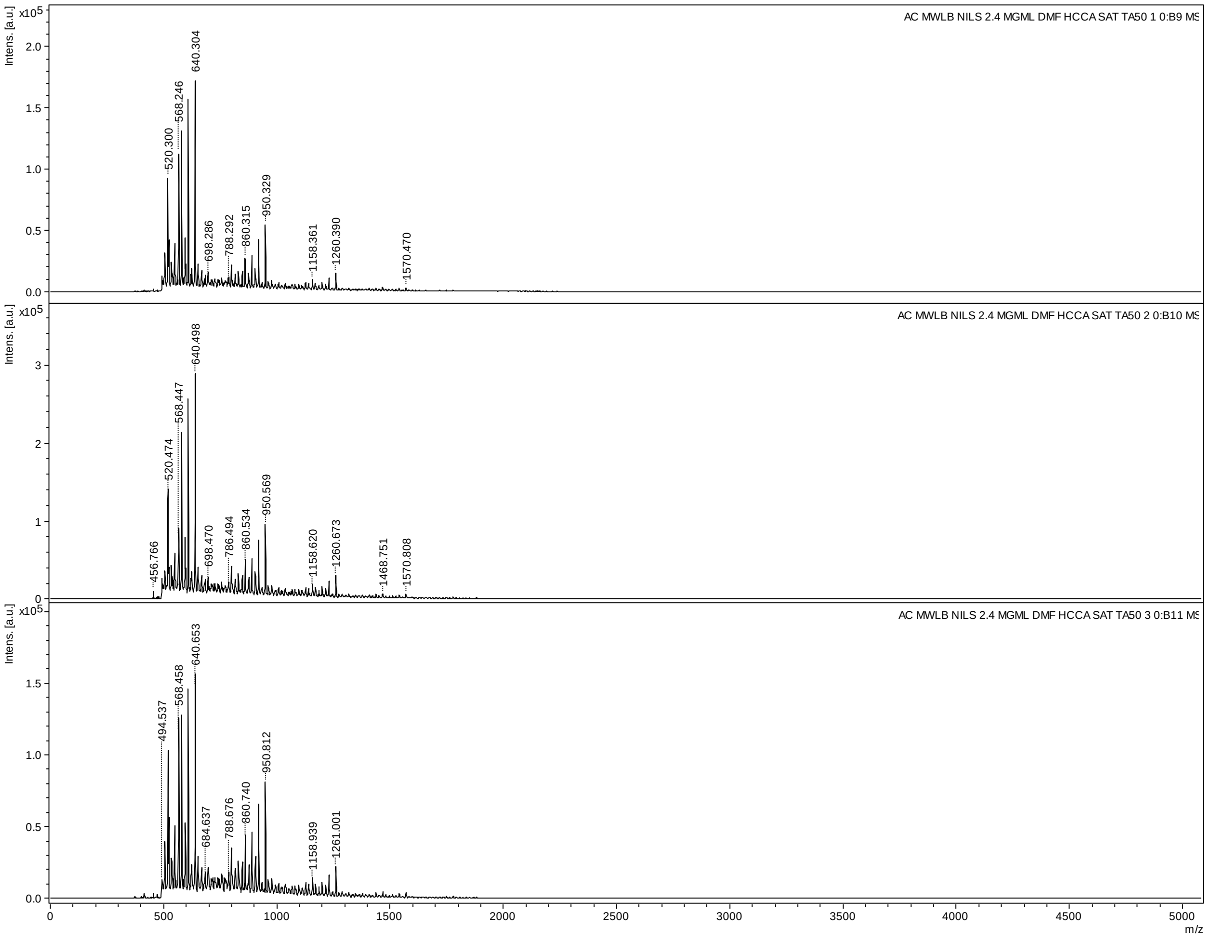

Figure 10. MALDI-TOF MS spectra of acetylated milled wood lignin from Birch using saturated HCCA in TA50 as matrix**.** Region 0-5000 m/z has been investigated using positive mode with reflectron. Y-axis displays signal intensity (arbitrary unit) and x-axis displays mass-over-charge (m/z).

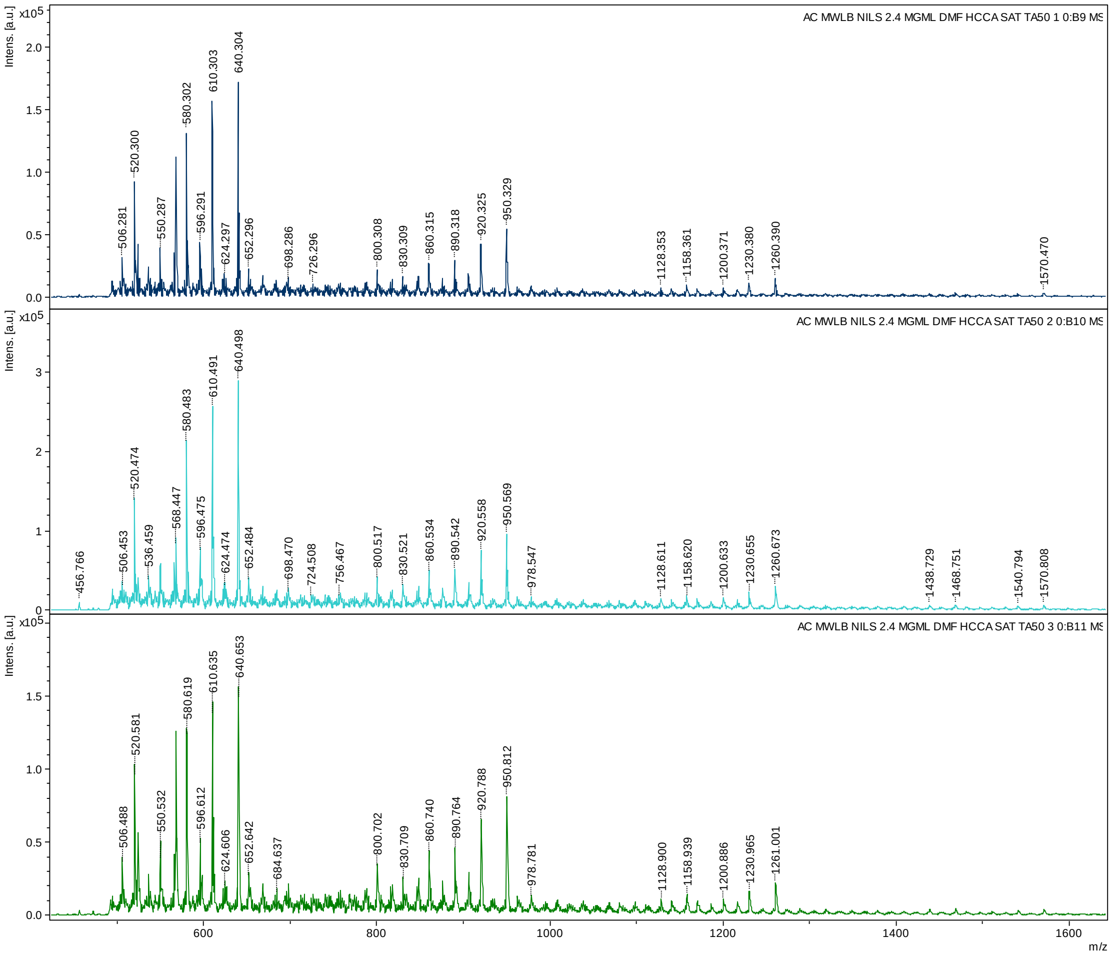

Figure 11. Expansion of 450-1600 m/z region of MALDI-TOF MS spectra on acetylated milled wood lignin from Birch using saturated HCCA in TA50 as matrix. Y-axis displays signal intensity (arbitrary unit) and x-axis displays mass-over-charge (m/z).

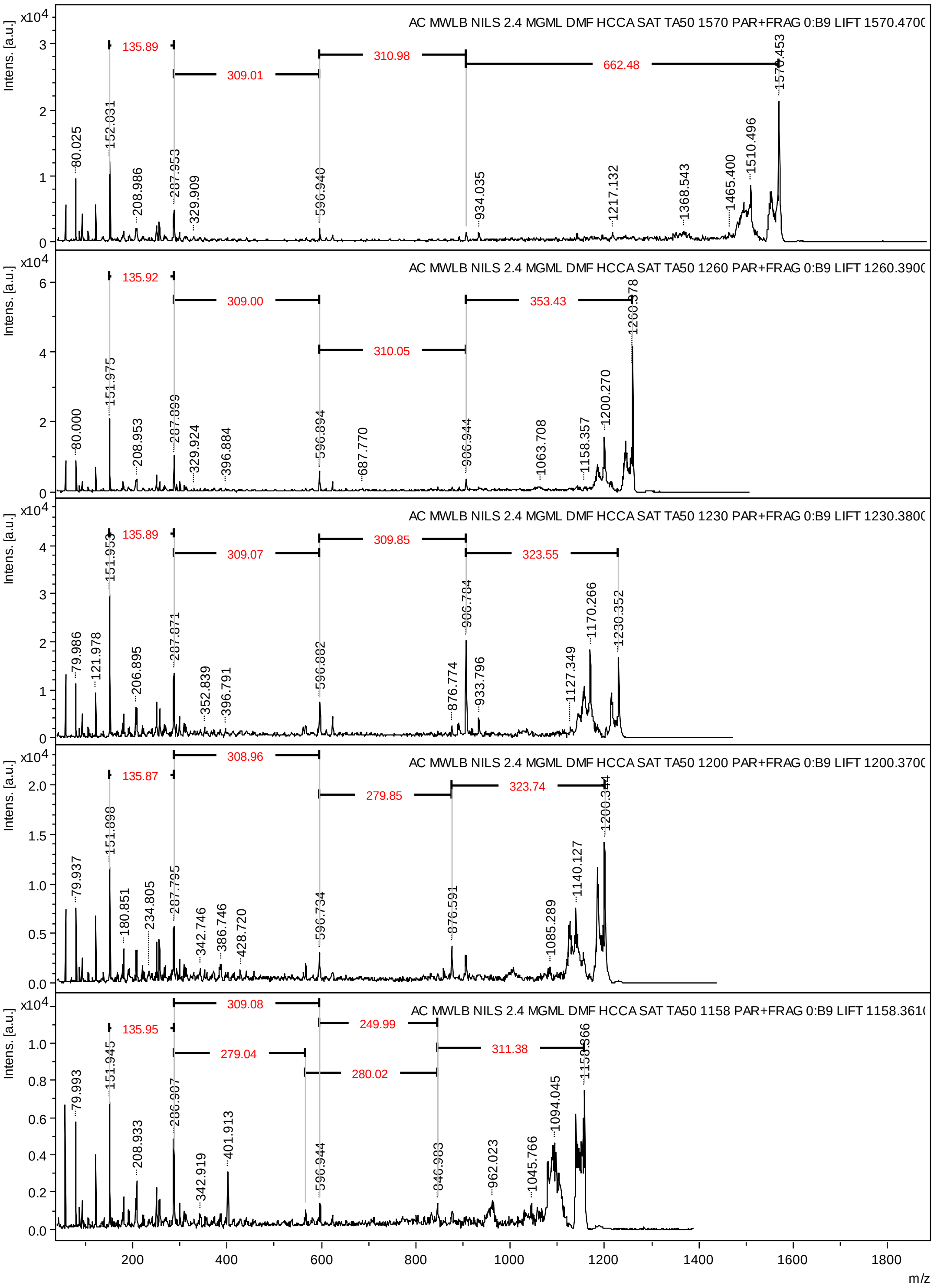

Figure 12. MALDI-LIFT-TOF/TOF spectra of adducts 1570, 1260, 1230, 1200, and 1158 m/z in acetylated milled wood lignin from Birch using saturated HCCA in TA50 as matrix. Y-axis displays signal intensity (arbitrary unit) and x-axis displays mass-over-charge (m/z).

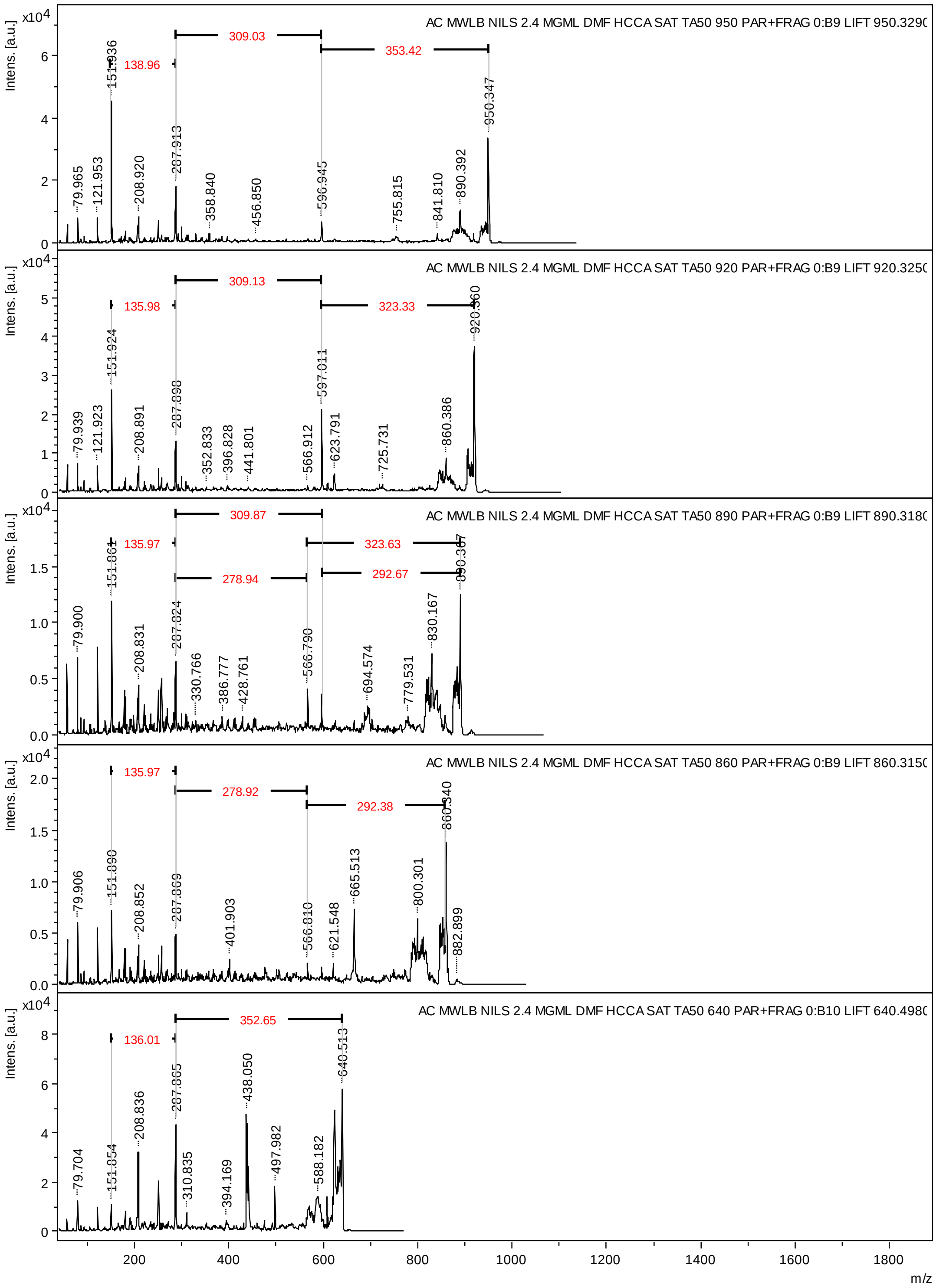

Figure 13. MALDI-LIFT-TOF/TOF spectra of adducts 950, 920, 890, 860, and 640 m/z in acetylated milled wood lignin from Birch using saturated HCCA in TA50 as matrix. Y-axis displays signal intensity (arbitrary unit) and x-axis displays mass-over-charge (m/z).

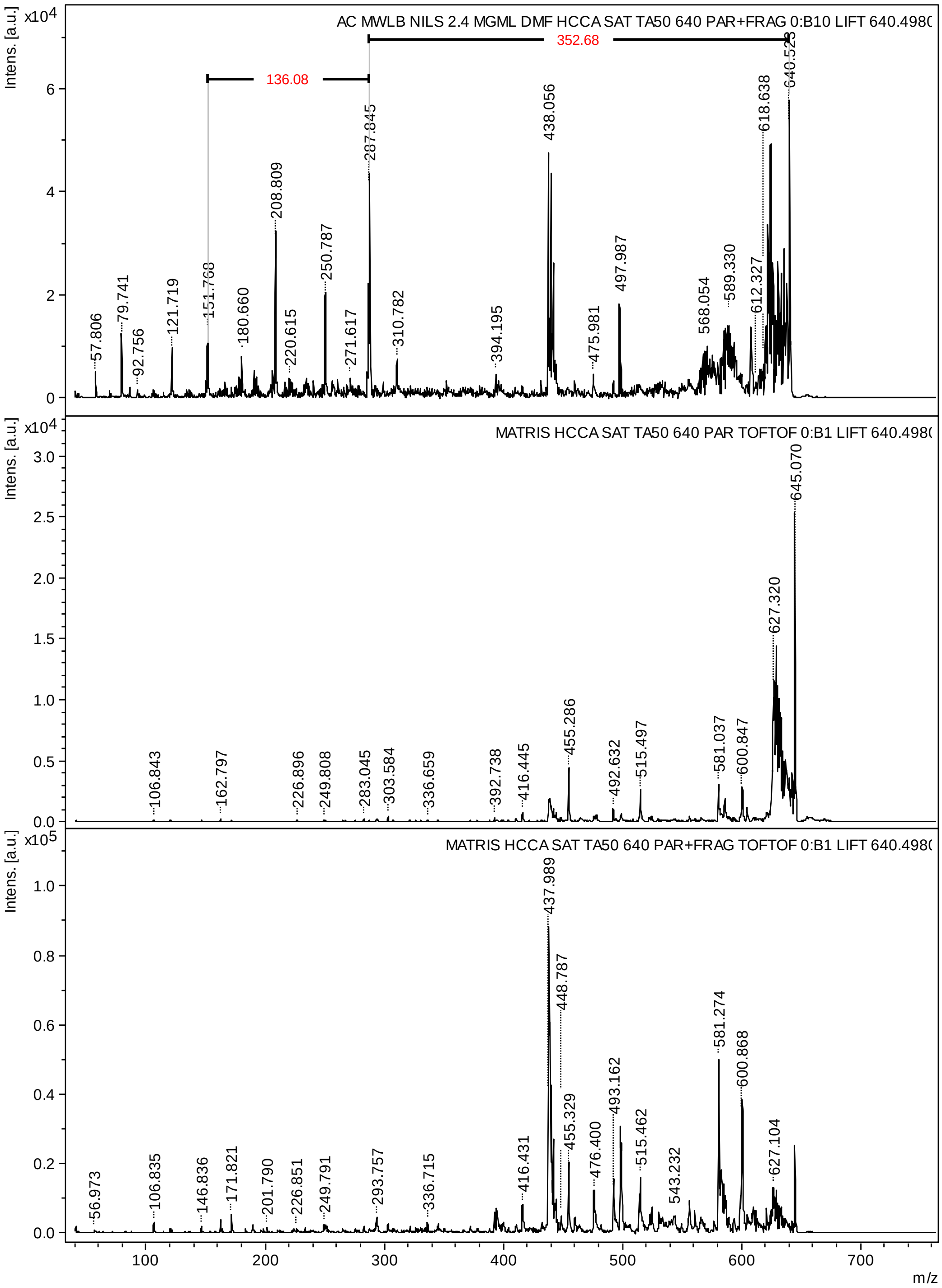

Figure 14. MALDI-LIFT-TOF/TOF spectra of adduct 640 m/z in milled wood lignin from Birch, compared to only parent and parent together with fragment spectra collected for pure HCCA matrix at matrix adduct found closest to 640 m/z.

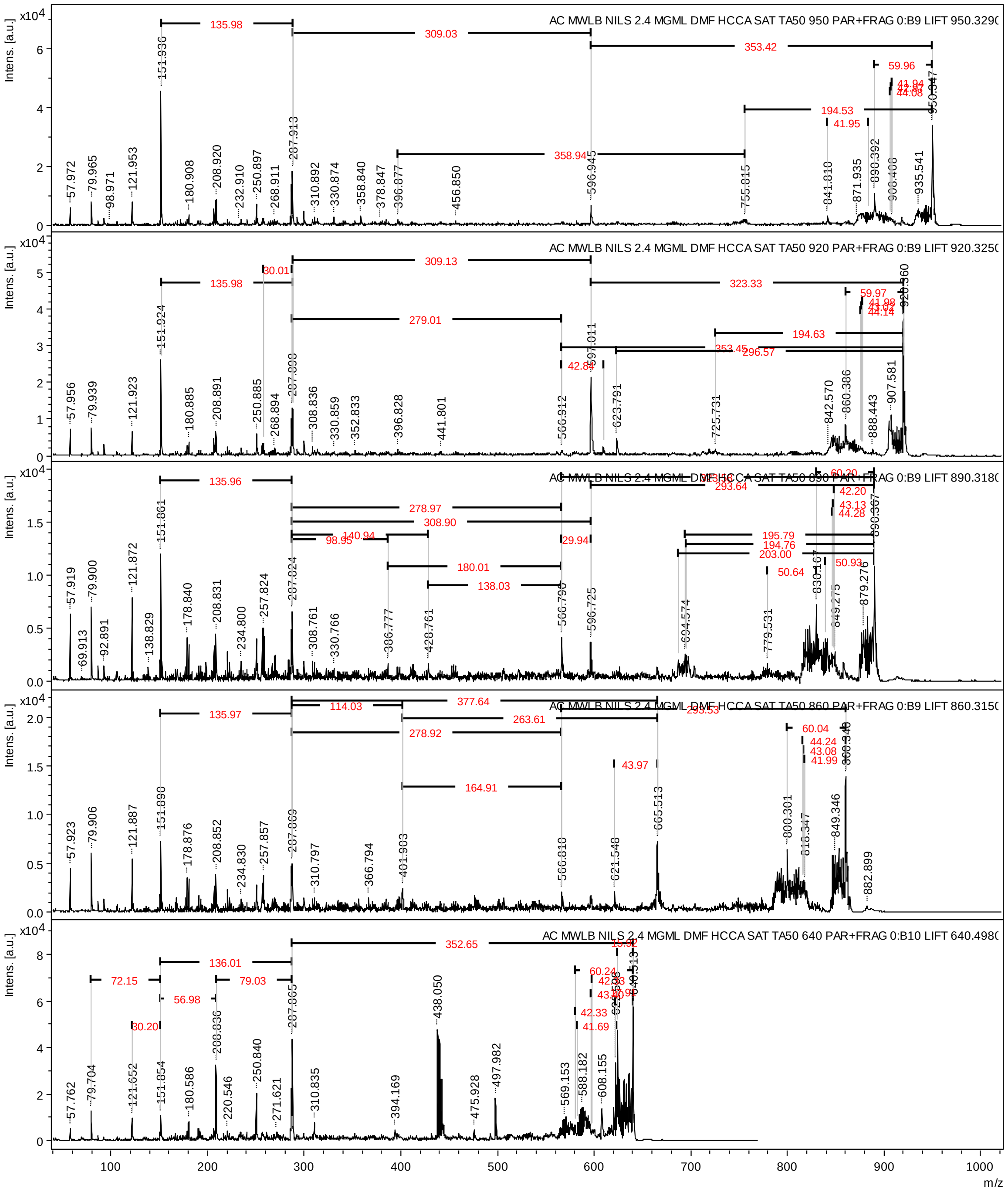

Figure 15. Expansion of MALDI-LIFT-TOF/TOF spectra of adducts 950, 920, 890, 860, and 640 m/z in acetylated milled wood lignin from Birch with additionally marked increments. Y-axis displays signal intensity (arbitrary unit) and x-axis displays mass-over-charge (m/z).

3. Postulated structures derived from MS^2

640 m/z

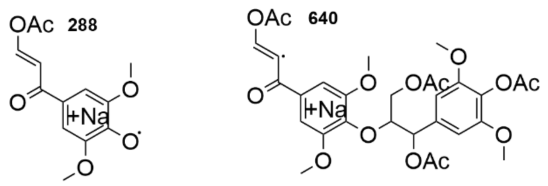

Figure 16. Postulated parent and fragment structures from MALDI-LIFT-TOF/TOF spectra of 640 m/z.

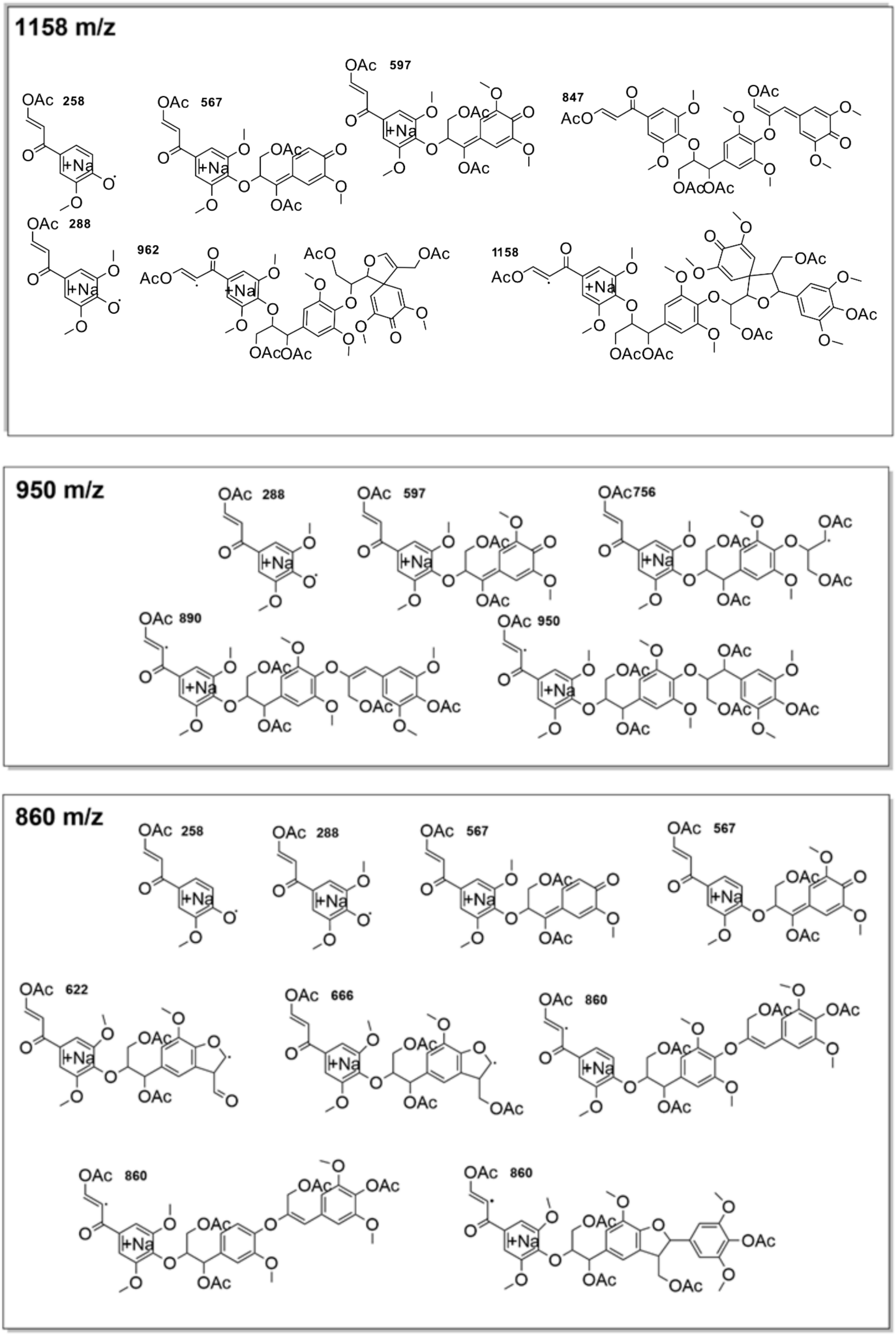

Figure 17. Postulated parent and fragment structures from MALDI-LIFT-TOF/TOF spectra of 1158, 950, and 860 m/z.

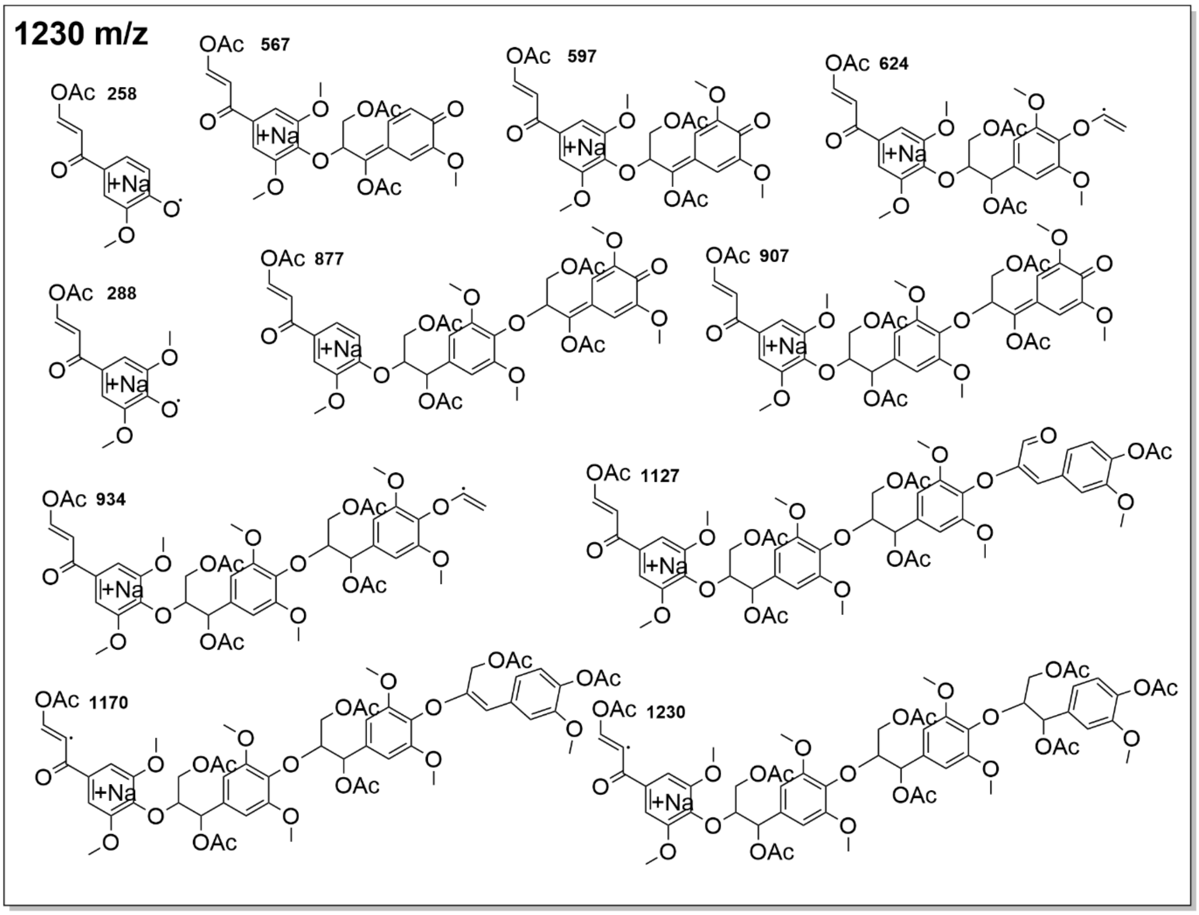

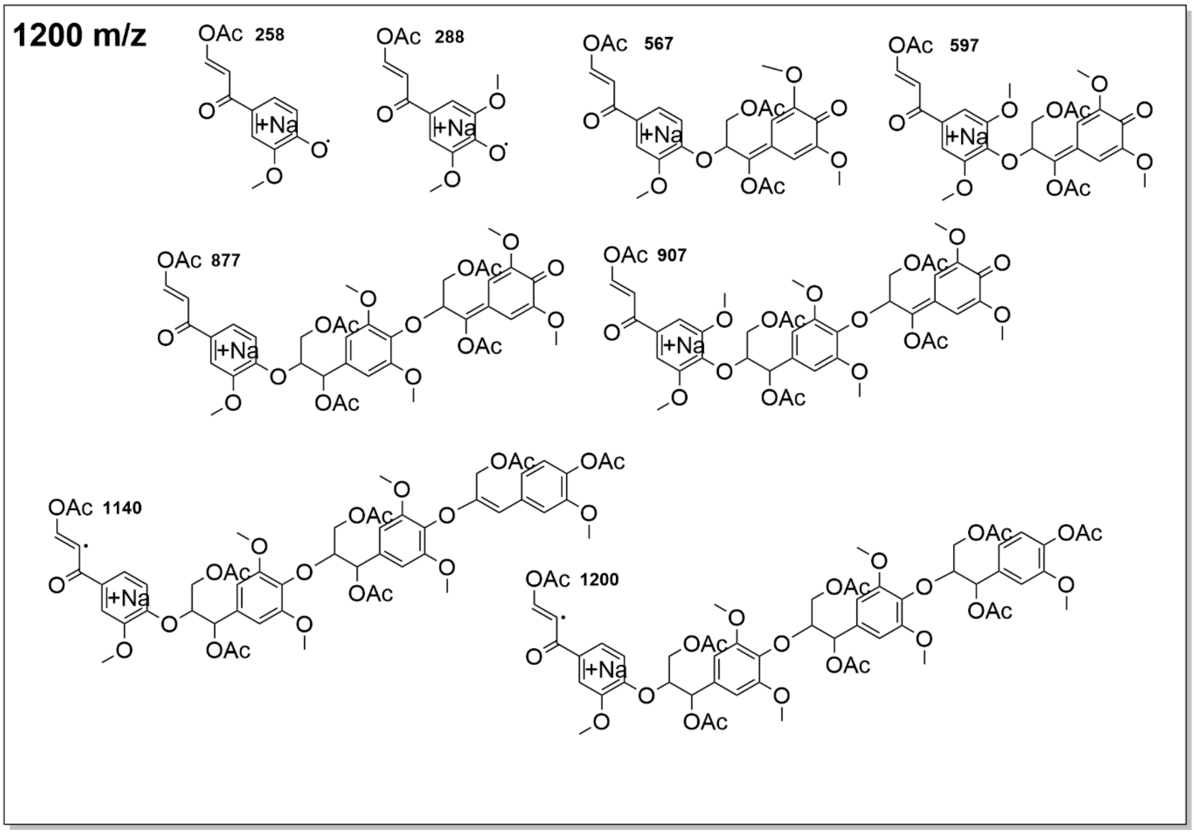

Figure 18. Postulated parent and fragment structures from MALDI-LIFT-TOF/TOF spectra of 1230 and 1200 m/z.

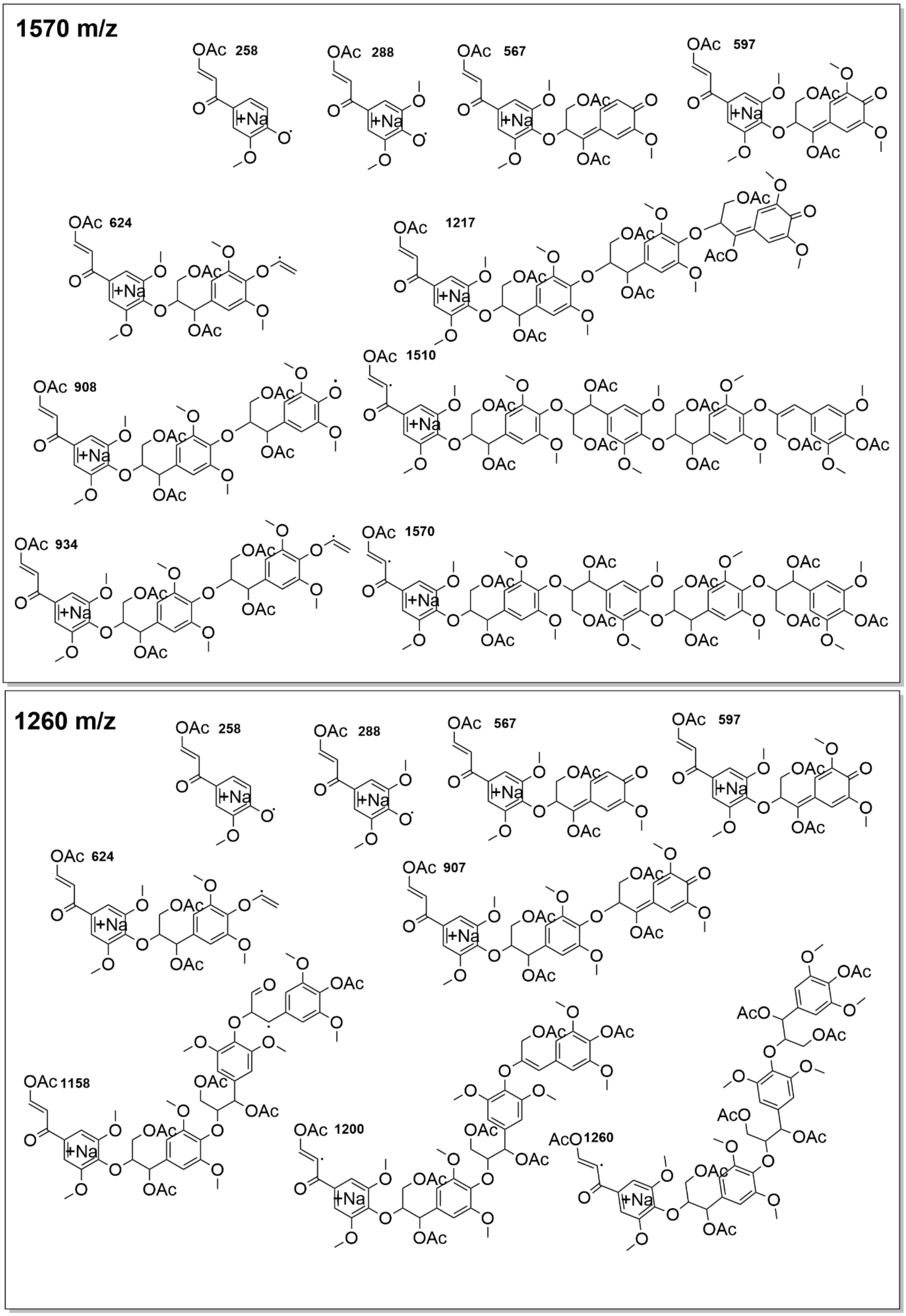

Figure 19. Postulated parent and fragment structures from MALDI-LIFT-TOF/TOF spectra of 1570 and 1260 m/z.

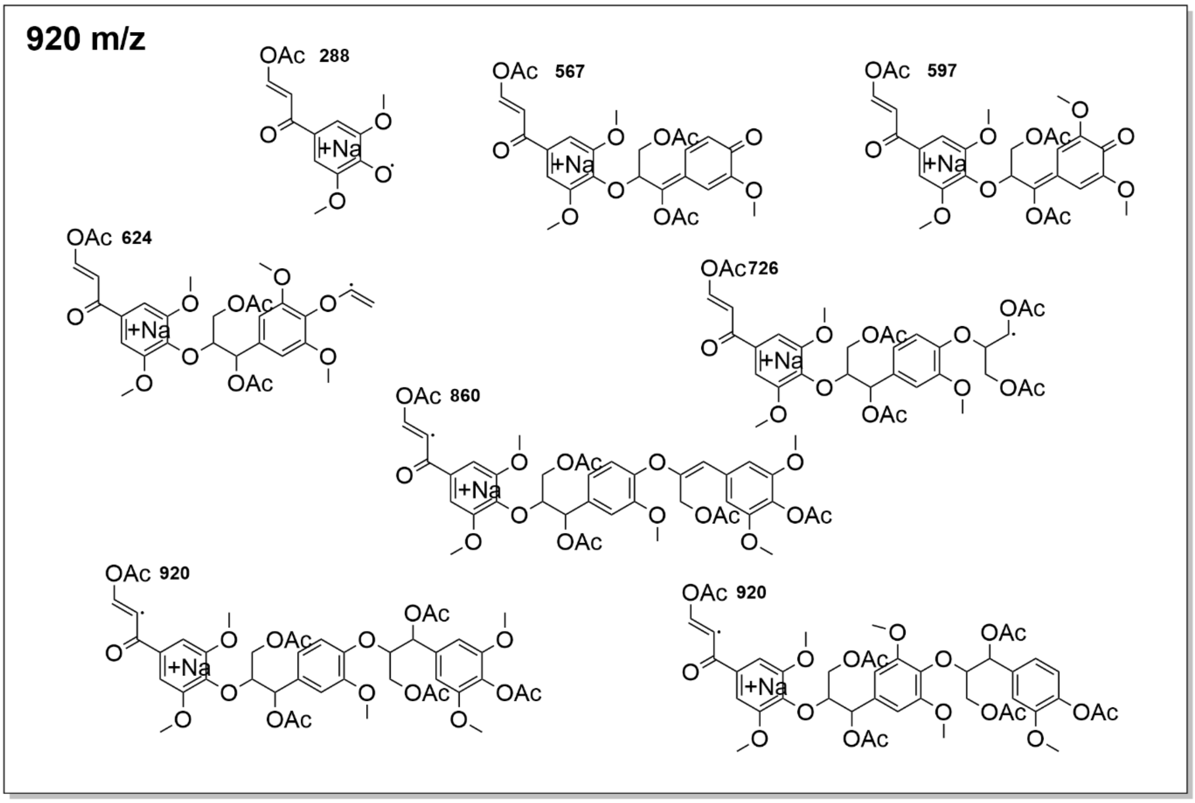

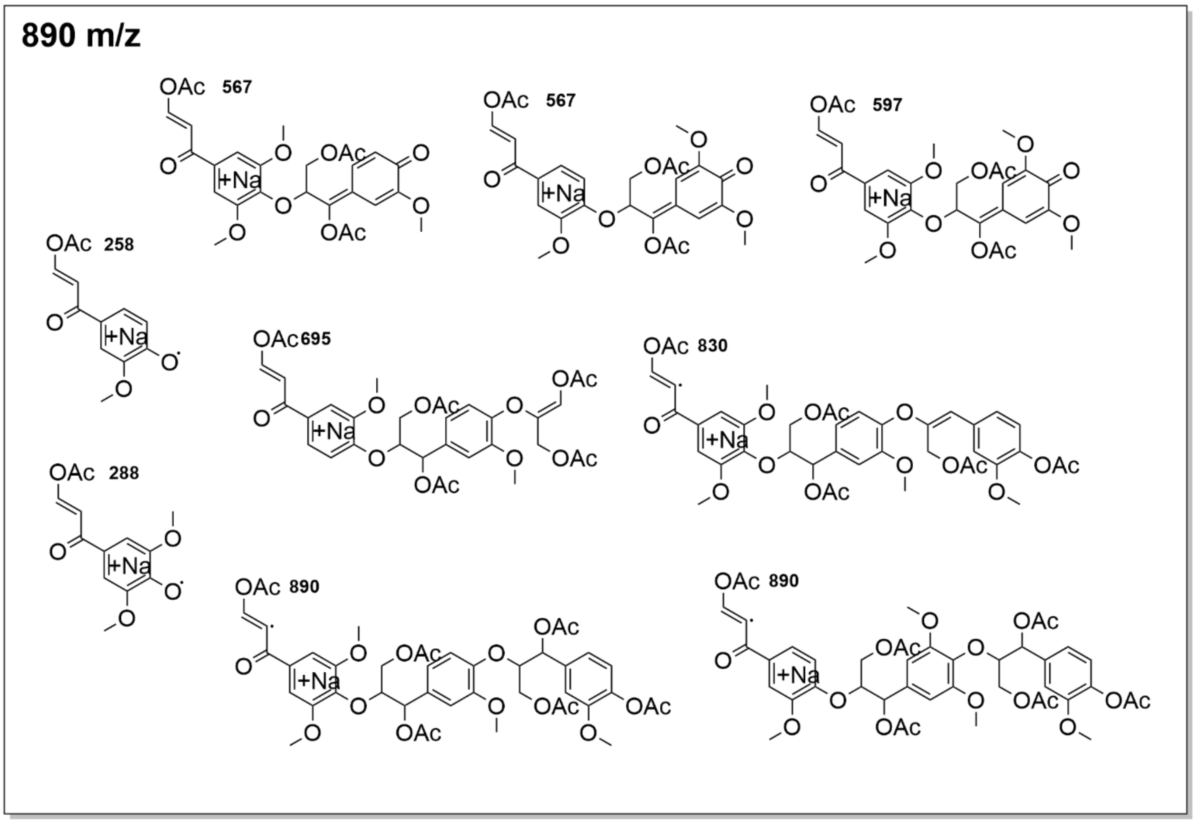

Figure 20. Postulated parent and fragment structures from MALDI-LIFT-TOF/TOF spectra of 920 and 890 m/z.

4. Statistical analysis

Table 1. Statistical analysis of peaks used to create the linkage progression maps.

						m/z			S/N		
Sample						average	sd	rsd	average	sd	rsd
1		2		3		da	da	%	a.u	a.u	%
m/z	s/n	m/z	s/n	m/z	s/n	m/z	m/z	m/z	s/n	s/n	s/n
579,930	333,000	579,942	103,000	579,942	144,000	579,938	0,006	0,001	193,333	100,168	51,811
609,945	395,000	609,956	121,000	609,957	175,000	609,953	0,005	0,001	230,333	118,506	51,450
639,959	410,000	639,972	120,000	639,971	179,000	639,967	0,006	0,001	236,333	125,141	52,951
860,033	88,000	860,048	25,000	860,051	39,000	860,044	0,008	0,001	50,667	27,010	53,310
890,044	95,000	890,059	26,000	890,061	42,000	890,055	0,008	0,001	54,333	29,488	54,273
920,053	145,000	920,068	36,000	920,071	62,000	920,064	0,008	0,001	81,000	46,483	57,386
950,062	198,000	950,078	45,000	950,081	80,000	950,074	0,008	0,001	107,667	65,454	60,793
1140,090	34,000	1140,112	13,000	1140,112	18,000	1140,105	0,010	0,001	21,667	8,957	41,339
1200,102	40,000	1200,120	15,000	1200,126	22,000	1200,116	0,010	0,001	25,667	10,530	41,027
1230,112	59,000	1230,127	22,000	1230,135	31,000	1230,125	0,010	0,001	37,333	15,755	42,201
1260,118	79,000	1260,133	26,000	1260,141	39,000	1260,131	0,010	0,001	48,000	22,554	46,987
800,012	65,000	1510,145	7,000	1540,158	10,000	1510,145	N/A	N/A	N/A	N/A	N/A
978,056	36,000	1540,153	9,000	1570,165	11,000	1540,156	0,002	0,000	9,500	0,500	5,263
1008,059	29,000	1570,155	10,000	800,030	31,000	1570,160	0,005	0,000	10,500	0,500	4,762
1038,069	31,000	800,029	21,000	978,074	17,000	800,024	0,010	0,001	39,000	18,833	48,289
1068,073	28,000	978,072	11,000	1008,077	15,000	978,067	0,008	0,001	21,333	10,656	49,951
1098,079	31,000	1008,077	10,000	1038,090	16,000	1008,071	0,008	0,001	18,000	8,042	44,675
1128,091	37,000	1038,091	11,000	1068,094	15,000	1038,083	0,010	0,001	19,333	8,498	43,957
1158,096	45,000	1068,094	11,000	1288,129	12,000	1068,087	0,010	0,001	18,000	7,257	40,318
		1288,126	9,000	1318,133	11,000	1288,128	0,001	0,000	10,500	1,500	14,286
		1318,128	9,000	1098,102	17,000	1318,131	0,003	0,000	10,000	1,000	10,000
		1098,101	12,000	1128,111	19,000	1098,094	0,011	0,001	20,000	8,042	40,208
		1128,109	13,000	1158,123	22,000	1128,104	0,009	0,001	23,000	10,198	44,339
		1158,113	15,000	1438,144	11,000	1158,111	0,011	0,001	27,333	12,815	46,884
		1438,134	9,000	1468,151	11,000	1438,139	0,005	0,000	10,000	1,000	10,000
		1468,143	9,000			1468,147	0,004	0,000	10,000	1,000	10,000

For all oligomers except a few up to 1260, values are an average of three found peaks. For 1510, a peak could be found only once. For peaks 1288, 1318, 1540, and 1570, peaks could be found twice. Values not being the sum of three measurements are highlighted in red.

For calculating average values (average), the built-in function “average” in excel has been used.

For calculating standard deviations (sd), the built-in function “stdev.p” in excel has been used.

Relative Standard Deviation (RSD) is calculated as following:

RSD = (sd) / (average) * 100%
